# Supplementary material for: Ageing and heterogeneity regarding autism spectrum conditions: a protocol paper of an accelerated longitudinal study
Source: BMJ Open. 2021 Mar 31;11(3):e040943. doi: 10.1136/bmjopen-2020-040943 (PMC8016100; doi:10.1136/bmjopen-2020-040943)
Supplement: Supplementary data [file bmjopen-2020-040943supp001.pdf]

Supplementary material

"Aging and heterogeneity regarding autism spectrum conditions: A protocol paper of an accelerated longitudinal study" Geurts, H.M., Agelink van Rentergem, J., Radhoe, T., Torenvliet, C., Putten Van der, W.J., Groenman, A.P.

Ethical approval:

Ethical approval for this study was obtained from the local ethical review board of the Psychology Department of the University of Amsterdam (Wave 1 2011-PN-1952 and 2013-PN-2668, Wave 2 2015-BC-4270, Wave 3 and 4 2018-BC-9285).
