# Supplementary material for: Ageing and heterogeneity regarding autism spectrum conditions: a protocol paper of an accelerated longitudinal study
Source: BMJ Open. 2021 Mar 31;11(3):e040943. doi: 10.1136/bmjopen-2020-040943 (PMC8016100; doi:10.1136/bmjopen-2020-040943)
Supplement: Author's manuscript [file bmjopen-2020-040943.draft_revisions.pdf]

BMJ Open

BMJ Open is committed to open peer review. As part of this commitment we make the peer review history of every article we publish publicly available.

When an article is published we post the peer reviewers' comments and the authors' responses online. We also post the versions of the paper that were used during peer review. These are the versions that the peer review comments apply to.

The versions of the paper that follow are the versions that were submitted during the peer review process. They are not the versions of record or the final published versions. They should not be cited or distributed as the published version of this manuscript.

BMJ Open is an open access journal and the full, final, typeset and author-corrected version of record of the manuscript is available on our site with no access controls, subscription charges or pay-per-view fees (http://bmjopen.bmj.com).

If you have any questions on BMJ Open's open peer review process please email info.bmjopen@bmj.com

BMJ Open

Senescence and heterogeneity regarding autism spectrum conditions: A protocol paper of an accelerated longitudinal study

Journal:	*BMJ Open*
Manuscript ID	bmjopen-2020-040943
Article Type:	Protocol
Date Submitted by the Author:	27-May-2020
Complete List of Authors:	Geurts, Hilde M.; University of Amsterdam, Psychology; Dr Leo Kannerhuis (Youz),<br>Agelink van Rentergem, Joost; University of Amsterdam, Psychology<br>Radhoe, Tulsi; University of Amsterdam, Psychology<br>Torenvliet, Carolien; University of Amsterdam, Psychology<br>Van der Putten, Wikke J; University of Amsterdam, Psychology; Dr Leo Kannerhuis (Youz),<br>Groenman, Annabeth P; University of Amsterdam, Psychology
Keywords:	Adult psychiatry < PSYCHIATRY, Old age psychiatry < PSYCHIATRY, Developmental neurology & neurodisability < PAEDIATRICS

SCHOLARONE™
Manuscripts

I, the Submitting Author has the right to grant and does grant on behalf of all authors of the Work (as defined in the below author licence), an exclusive licence and/or a non-exclusive licence for contributions from authors who are: i) UK Crown employees; ii) where BMJ has agreed a CC-BY licence shall apply, and/or iii) in accordance with the terms applicable for US Federal Government officers or employees acting as part of their official duties; on a worldwide, perpetual, irrevocable, royalty-free basis to BMJ Publishing Group Ltd ("BMJ") its licensees and where the relevant Journal is co-owned by BMJ to the co-owners of the Journal, to publish the Work in this journal and any other BMJ products and to exploit all rights, as set out in our licence.

The Submitting Author accepts and understands that any supply made under these terms is made by BMJ to the Submitting Author unless you are acting as an employee on behalf of your employer or a postgraduate student of an affiliated institution which is paying any applicable article publishing charge ("APC") for Open Access articles. Where the Submitting Author wishes to make the Work available on an Open Access basis (and intends to pay the relevant APC), the terms of reuse of such Open Access shall be governed by a Creative Commons licence – details of these licences and which Creative Commons licence will apply to this Work are set out in our licence referred to above.

Other than as permitted in any relevant BMJ Author's Self Archiving Policies, I confirm this Work has not been accepted for publication elsewhere, is not being considered for publication elsewhere and does not duplicate material already published. I confirm all authors consent to publication of this Work and authorise the granting of this licence.

For peer review only

Senescence and heterogeneity regarding autism spectrum conditions: A protocol paper of an accelerated longitudinal study

Geurts, H.M.[1,2] , Agelink van Rentergem, J.[1],

Radhoe, T.[1], Torenvliet, C. [1], Putten Van der, W.J.[1,2] , Groenman, A.P.[1]

[1]Dutch Autism & ADHD Research Center, Department of Psychology, University of Amsterdam, Amsterdam, the Netherlands

[2]Dr. Leo Kannerhuis, autism clinic (Youz/Parnassia Group), Amsterdam, the Netherlands

Address correspondence to:

Correspondence author information: Hilde M. Geurts, H.M.Geurts@uva.nl, phone +31 20 525 6843, Dutch Autism and ADHD Research Center (d'Arc), Brain & Cognition, Department of Psychology, University of Amsterdam, Postbus 15915, 1001 NK, Amsterdam, The Netherlands.

For peer review only

Number of words (excl. References and Tables): 4036

Number of Tables: 3

Number of Figures: 2

Appendix: No

Abstract

Introduction: Autism spectrum conditions (ASC) develop early in life and are thought to last a lifetime. However, ASC research has two major knowledge gaps that hinder progression in understanding the concept of ASC and in providing proper support for autistic adults: 1) the majority of knowledge about ASC mainly stems from childhood studies so little is known about older autistic adults; 2) while it is broadly recognized that ASC is a heterogeneous condition, we do not yet understand the differences in trajectories leading to their future outcome. We aim to fill both knowledge gaps.

Methods and analysis: A multistage overlapping cohorts design assessing (cognitive) aging in ASC, is designed to obtain an accelerated longitudinal dataset. Data, including a multitude of questionnaires, diagnostics, and cognitive tests, are collected over four waves within a 10-year time frame. This will provide information regarding actual changes in quality of life, co-occurring health conditions, and cognition as well as the possibility to test external validity and temporal stability in newly formed behavioral subtypes. Participants consist of three groups of adults aged 20 to 90 years: 1) with a clinical diagnosis of ASC; 2) with a clinical diagnosis of ADHD, but no ASC; 3) no ASC/ADHD (i.e., comparison group). The sample size differs between waves and instruments. Detailed analysis plans will be preregistered in AsPredicted.

Ethics and dissemination: Ethical approval for this study was obtained from the local ethical review board of the Department of Psychology of the University of Amsterdam (Wave 1 2011-PN-1952 and 2013-PN-2668, Wave 2 2015-BC-4270, Wave 3 and 4 2018-BC-9285). In line with the funding policies of the grant organization funding this study, future papers will be published open access.

KEY WORDS: Autism, Aging, Heterogeneity, Cognition, Protocol, Longitudinal

For peer review only

Strenghts and limitations of this study

Strenghts are:

1. This study has an accelerated longitudinal design, so in a relative short time frame for the first time actual age-related (cognitive) changes can be assessed in older autistic adults.
2. We will use in-depth phenotyping due to inclusion of measures of known vulnerability and protective factors regarding aging and a wide range of diagnostic instruments, questionnaires, and cognitive tests.
3. The external validity and temporal stability of behavioral ASC subgroups can be tested, which are both aspects that are often not implemented in subgroup study designs.

Limitations are:

1. The first two measurement waves were not set up as being part of an accelerated longitudinal design which led to high attrition rates.
2. As such, not all measures are the same across all four measurement waves.

For peer review only

Introduction

Neurodevelopmental conditions, like an autism spectrum condition (ASC), are defined as conditions with early atypical brain development, which have cascading effects on cognitive processes important during the entire lifespan [1]. At the core of the Diagnostic and Statistical Manual of Mental Health Disorders Fifth Edition (DSM-5) ASC diagnosis are difficulties in social interaction and communication, sensory sensitivities, and stereotyped or repetitive behaviors and interests [1]. A conservative estimate is that one in hundred persons meets the criteria [2], but recent estimates seem to be higher and vary widely [3]. Whereas ASC was originally perceived as a childhood condition, which was often comorbid with a severe intellectual disability and had an overall poor outcome, we now know that ASCs are not restricted to childhood or to those with low intellectual functioning, and that there is large heterogeneity across persons and across time in type and severity of the disabilities experienced [4,5]. This led to the idea that while ASC is a behaviorally defined condition, it likely consists of different conditions with diverse outcomes which are potentially falsely lumped together in one diagnostic category [6,7]. Understanding the concept, course, and consequences of ASCs is critical in order to develop appropriate support for (old) autistic[1] adults and for adults with related neurodevelopmental conditions. Unfortunately, ASC research hardly exceeded young adulthood, so what happens in old age remained exempt of empirical scrutiny [8,9]. Thus, two questions need to be answered:

1. How do people with ASCs progress in late adulthood?
2. How can we understand the differences among people with ASC in future outcome?

A dominant assumption is that autistic adults are at a larger risk for accelerated cognitive aging [10,11]. However, recent findings from cross-sectional studies suggest that this assumption is

[1] We chose to use the term autism spectrum conditions as the traditional and official DSM-5 term "autism spectrum disorder" and we use identity first language as this is what a majority of autistic adults seem to prefer in the UK. We are well aware that in other countries, like the Netherlands, the preference of autistic adults is not to use identity first language, but given that this paper is written in English we chose the UK preference.

For peer review only - http://bmjopen.bmj.com/site/about/guidelines.xhtml

neither appropriate for all cognitive domains nor appropriate for all autistic adults. We, for example, found that while autistic adults reported that they are experiencing a large number of cognitive challenges in daily life, these autistic adults hardly differed from controls on a wide range of cognitive domains (e.g., episodic verbal memory, inhibition [12–14]) and not all adults experience the same cognitive challenges [14]. The ASC group performed worse with respect to advanced social knowledge (i.e., mentalizing) and executive functioning (especially the ability to come up with various adequate solutions when confronted with a challenge, i.e., generativity [14]). But, interestingly, the observed effect sizes were small and only a small proportion of the autistic adults seem to have clinically relevant cognitive problems. In studies including autistic children, typically medium effect sizes are observed and performance differences are apparent across a broader range of cognitive domains [15]. Moreover, independent empirical studies including old autistic individuals recently reported similar findings as Lever and colleagues observed [16,17]. So, in sharp contrast to the dominant hypothesis, these novel findings suggest that with increasing age, the cognitive differences between autistic adults and non-autistic adults become less pronounced. Yet, even more challenging for the dominant view, is that strong age-related cognitive differences are found in non-autistic adults, with respect to episodic visual memory and visual working memory, but *not* in autistic adults [12,14]. Also, while across different ages, autistic adults seemed to perform worse with respect to mentalizing, performances of adults with and without an ASC diagnosis did not differ in those over 50 years [14]. Hence, recent findings concerning cognition across adulthood are not just in contrast with the accelerated aging hypothesis, but could even suggest that autistic adults are less sensitive to the typical cognitive aging effects. This is why we proposed an alternative hypothesis, which is that the cognitive strategies (a subgroup of) autistic adults use, while disadvantageous when young, are beneficial (i.e., protective) when older [10,14]. However, in order to test such a hypothesis, we first need to

For peer review only - http://bmjopen.bmj.com/site/about/guidelines.xhtml

determine whether previous cross-sectional findings can be replicated cross-sectionally as well as longitudinally, in sufficiently large groups. This is especially of importance as recently various small studies have been published with rather mixed findings regarding which cognitive functions are predominantly associated with specific cognitive aging patterns [17–21].

Moreover, we need to determine whether we can actually form well-validated adult ASC subtypes. If there are indeed adulthood subtypes, both aging hypotheses (i.e. accelerated cognitive decline and protective cognitive strategy use) can be true for different subtypes in different cognitive domains. Ideally, when defining subtypes, one uses well-accepted low-cost, nonintrusive measures such as short questionnaires [22]. Behaviorally defined subgroups, so far, differed mainly in severity (i.e., quantitative instead of qualitative differences; [23]). However, it could well be that valid subtypes can be formed when symptom information is combined with other important behavioral characteristics, which are in the general population considered to be vulnerability (e.g., stressful life events and lack of control) and protective factors (e.g., physically activity and a sound social network) for a wide range of outcome measures.

These adulthood subtypes are not just of relevance for cognitive aging, but are also of relevance to gain more tailored information regarding someone's prognosis. Such newly formed subtypes need to be tested by checking multiple external validators, such as temporal stability and clinical outcome (next to change in cognitive abilities also the presence of comorbidities, subjective wellbeing) at follow up. Ideally, in order to test the specificity of newly formed subtypes, another clinical group needs to be included. Put differently, including data from an additional neurodevelopmental condition (like ADHD) ensures that we can test whether the obtained subgroup findings will be specific for ASCs and follow the distinction between ASC and other neurodevelopmental conditions or whether the obtained subtypes cross

For peer review only

the classical borders between these conditions. Given the overlap between ASC and ADHD [24], the second scenario is more likely. Hence, the proposed study will not just gain relevant knowledge for those with an ASC diagnosis, but will also add to the understanding of aging in related neurodevelopmental conditions.

In sum, we have two major aims;

1. To arbitrate between contrasting ASC-related cognitive aging hypotheses (respectively, accelerated cognitive decline and protective cognitive strategy use) by studying aging trajectories;
2. To determine (late) adulthood ASC subtypes and to explore if there are specific prognostic behavioral markers that are related to the observed heterogeneity, and can be used in clinical practice to predict who might or might not be prone to a) co-occurring mental health conditions; b) lower quality of life; and c) cognitive decline.

We hypothesize that on overall group level we will replicate the findings of our cross-sectional study [12,14], cross-sectionally as well as longitudinally. This implies that for the majority of cognitive domains, the aging trajectory of autistic adults will parallel the aging trajectory of the controls, but for visual memory, visual working memory, and Theory of Mind the aging pattern would be in line with the protective cognitive strategy hypothesis. Moreover, we hypothesize that based on known vulnerability and protective factors for typical accelerated (cognitive) aging next to ASC symptom measures, valid ASC subtypes can be determined. However, currently there are no longitudinal datasets focusing on autistic adults which include a) a significant proportion of adults over 55 years of age, b) sufficiently detailed measures on (mental health), quality of life, and objective and subjective cognition; c) comparison groups with and without other neurodevelopmental conditions (i.e., ADHD);

For peer review only

and d) a sufficiently large sample. Each of the criteria needs to be met in order to properly test the aforementioned hypotheses. Moreover, earlier studies often did not consult autistic adults in the research process while the potential outcomes of these study are especially relevant to them. Therefore, we designed the current accelerated longitudinal study with regular input from autistic adults.

Thus, in this study we will map changes across time in cognition, mental health, and subjective well-being in autistic adults and non-autistic adults in mid and late adulthood (30 to 90 years). This way we can test whether we can replicate earlier cognitive findings and we can form behaviorally defined subtypes in autistic adults for which we can test specificity, external validators, temporal stability, and predictive validity.

Methods

Study design

This study follows a multistage overlapping cohorts design assessing (cognitive) aging in ASC, designed to obtain an accelerated longitudinal dataset. This protocol paper concerns four waves which encompass three cohorts of people. Please note that in Wave 1 and 2, we did not have funding yet for an accelerated longitudinal study. This means that these Waves were originally set up as independent studies. Currently, we are in the third wave of assessments (May 2020). The time frame of data-collections is as follows: Wave 1 March 2012 until July 2014 (Cohort 1); Wave 2 December 2015 to December 2016 (Cohort 2); Wave 3 September 2018 until +/- July 2020 (Cohort 3); Wave 4 November 2020 until +/- July 2021. At the time of writing the current protocol, the COVID-19 pandemic happened. This will likely affect the exact timing of the Wave 3 and 4. but currently we do not know the

For peer review only

exact consequences. Please see Figure 1 and 2 for a visual depiction of the different waves and cohorts.

-Insert Figure 1 here-

Procedure and setting

After potential participants showed interest in participation, they received a study information package. Only when we received informed consent, people were actually included in the study. Please note that participants of Wave 1 were originally recruited for a cross-sectional study only, but the majority was asked to indicate whether we could contact them for future studies. Approximately 80% of the ASC group and approximately 40% of the COM group granted such permission. The Wave 1 participants between 30 and 80 years of age who gave an affirmative answer were partly recruited for Wave 2, but again for this specific Wave only. Of the newly recruited participants at Wave 2 approximately 90% of the ASC group and approximately 65% of the COM group granted permission to contact them for future studies. At Wave 3 participants were asked to participate in both Wave 3 and 4, meaning that they needed to give consent to take part in two measurements at two distinct time-points with a one- to two-year interval. In each Wave participants received a series of questionnaires (on paper or via internet, see Table for details). The first selection (see inclusion/exclusion criterion) is based on the first series of questionnaires (e.g., socio-demographics, diagnostic information, Autism spectrum Quotient (AQ), AD(H)D-SR)) participants filled out. If participants meet the inclusion criteria for the subtyping study, they fill out the second series of questionnaires. When participants also meet the first series of inclusion criteria for the cognitive study (Wave 1, 3, and 4), they are invited for a face-to-face session at the university, or closer to the participant’s home upon request. In this first face-to-face session,

For peer review only

the Autism Diagnostic Observation Scale (ADOS or ADOS-2; T1) or *Nederlands Interview ten behoeve van Diagnostiek Autismespectrumstoornis bij volwassenen* (Dutch Interview for assessment of autism spectrum disorders in adults; NIDA; T2), the International Neuropsychiatric Interview (MINI), Mini Mental State Examination (MMSE), and Wechsler Adult Intelligence Scale (WAIS) subtests are administered when it concerned autistic adults, but the ADOS(-2) (or NIDA) is not administered in the comparison groups. In Wave 3 and 4, a questionnaire called the menopause rating scale (MRS) is added to this first session in a subset of the participants. If the autistic participants still meet inclusion criteria, a second face-to-face session will take place in which the cognitive tests will be administered (see for the specific tests Table 2). For the non-autistic/non-ADHD comparison (COMP) group the two face-to-face sessions were combined on request. This implies that in theory in this group it could be that subgroup of COMP participants did not meet the inclusion criteria for the cognitive section of the study, but still did do the cognitive tasks. In each Wave where cognitive testing is part of the protocol, there were 16 predetermined test orders which are counterbalanced across participants. Both sessions took place in a quiet distraction-free room in one or two sittings (each approximately 2 hours). In Wave 1 control participants received a small additional fee for participating. In Wave 3 and 4 all participants will also receive additional financial compensation for filling out the questionnaires as well as the face-to-face sessions (max. 30 euros). In all Waves travel costs are reimbursed. Some participants of Wave 1 were also invited to take part in an MRI study [25–27], but that study is not included in this protocol.

Study population and recruitment

For Wave 1 individuals between 18 and 80 years were recruited, for Wave 2 and onwards individuals between 30 and 90 years were recruited. Individuals with ASC and/or ADHD

For peer review only

were recruited through several mental health institutions across the Netherlands, by means of advertisement on client organization websites and newsletters, and through social media (i.e., Twitter and LinkedIn). Comparison participants were recruited through social media and the personal networks of the researchers and participants.

Inclusion criteria for all participants were: 1) no intellectual disability, as reported on questionnaires, or an IQ >70 as estimated using two subtests of the Wechsler Adult Intelligence Scale III or IV-NL (WAIS-III; [28] WAIS-IV-NL; [29,30]); and (2) sufficient understanding of Dutch language (i.e. at least one Dutch parent, Dutch was spoken in their family or when participants fill in more than 90% of the questionnaires).

An addition inclusion criterion for the ASC or ADHD group wasa prior clinical ASD and/or ADHD diagnosis according the DSM- IV or DSM-5 [1,31]. For COMP group the exclusion criteria were: 1) clinical diagnosis of ASC or ADHD in themselves or direct family members; 2) an elevated score on the AQ >32 (AQ; [32] or an elevated score on ADHD-SR ≥ 6 [33]; 3) more than 1 psychosis.

For cognitive testing additional inclusion criteria were applied. All participants have to have 1) MMSE >18 [34]]; 2) no history of neurological disorders (e.g., epilepsy, stroke, cerebral contusion); 3) no current alcohol or drugs dependency; and 4) no more than 1 psychosis.

Measures

All measures and the waves at which they were administered are depicted in Tables 1 through 3.

Questionnaires.

For peer review only

First a series of general questions were asked regarding, among others, birth year and month, gender, education, diagnostic history, ASC/ADHD in family members, and co-occurring conditions. Second, standard questionnaires are included which assessed several domains of functioning, namely: a) ASC domains including general autism traits [AQ;[32,35]], interpersonal reactivity [IRI; [36,37]], sensory sensitivities [SSQ;[14,38]]; b) mental health including ADHD characteristics [ADHD-SR; [33]]; general mental health problems (SCL-90; [39,40]; c) Physical health [Health questionnaire; [41]] and physical activity [IPAQ; [42]]; d) Well-being [Happiness; [43]] and Quality of life [WHOQoL-Bref; [44,45]]; e) Stress and worries [Worry Scale/ Fear Questionnaire; [46,47]] and Stress and self-control [Pearlin Mastery Scale; [48]]; f) subjective cognitive complaints [CFQ; [49,50]]; g) important life events [Brugha;[51] [52]]; h) emotions [PANAS; [53,54]; i) social network [Close Persons Questionnaire (CPQ); [55]]; j) camouflaging of autistic traits [CAT-Q; [56,57]]; h) menopausal complaints [MRS;[58]].

-Insert Table 1 around here-

Diagnostic assessment

Global cognitive functioning was assessed with the MMSE; [34][59] and general intelligence was estimated by two subtests [Vocabulary and Matrix Reasoning] of the WAIS-III Wave 1/WAIS-IV Wave 3 and 4 [28][29,30]). Autistic traits were assessed with either the ADOS [60,61] or the NIDA [62], dependent on the measurement the participant was performing (i.e. ADOS was administered at the first measurement moment (T1) for each of the autistic participants and the NIDA at subsequent moments (T2). Psychiatric comorbidity was assessed with the MINI [63,64] in all participants independent of group.

For peer review only

-Insert Table 2 around here-

Cognitive assessment

Participants completed a comprehensive battery of cognitive assessments in the following domains; a) Memory, including Episodic verbal memory [Rey auditory verbal learning task (RAVLT); [65]], Episodic visual memory [Wechsler memory test- III (WMS-III; [66])], Prospective memory [Amsterdam breakfast task;[67,68] in-house development author AG], Working memory [N-Back; [12]]; b) Social cognition [Faux pas; Theory of Mind;[69,70]]; c) Processing speed [2-choice reaction time (2-CRT)[14]];d) Executive functioning including generativity [Dutch version (DAT) of the Controlled Word Association Test (COWAT) [71]], planning [Tower of London; [72]], Inhibition [Simon task [13], Go/Nogo task;[in-house development by AGL], Flexibility [trail making test (TMT) A&B; [73]];d) attention (oddball; in-house development by AGL).

-Insert Table 3 around here-

Data management and quality control.

Following the institutional and national guidelines the data was pseudonymized and encrypted during and after data collection. For quality control the data of all Waves was entered by participating master students and research assistants and between 10-100% was checked by other master students, research assistants, and the PhD students involved in Wave 1, 3, and 4. The initial percentage checked depended on who entered the data for the first time and whether mistakes were discovered. When mistakes were discovered in the first checked subset, there was a 100% check.

Patient and Public Involvement statement

For peer review only

Before the start of Wave 3, we formalized the involvement of the major stakeholder group, the autistic adults. We meet a group of four older autistic adults at least three times a year for a four-year period in which we discuss, for example, study designs, questionnaires, letters, tasks and task instructions, study results, and the dissemination of these results. They are paid for their contribution. In earlier waves the involvement of the stakeholders was not formally organized.

Data analysis plan and Power.

The analyses of Wave 1 are described in detail in the published papers [12–14,74,75]. All data analysis plans regarding future manuscripts based on the combined data across the different waves will be preregistered in detail on AsPredicted or similar platforms before data collection ends. Between July 2019 and May 2020, we submitted the first six detailed analyses plans to AsPredicted.

The power analysis is based on Aim 1 (cognition). In order to detect a (continuous) change with these statistical methods on the included cognitive measures, we will need a sample size of +/- 100 participants in the ASC group and 85 participants in the COM group at two time points when assuming an effect size of .20 and power of .80 [76,77]. In order to reach at least these numbers we will need to recruit more T1 participants than needed for these analyses to a) be able to match on age and gender; and b) to assure sufficient participants at T2 when considering attrition rates of 30 (ASC group) to 50% (COM group). The inclusion criteria (see earlier) are the strictest for the cognitive study, which implies we need to oversample when collecting the questionnaire data. Required sample sizes for Aim 1 and 2 differ. For Aim 2 (subtyping) it is of importance that we include a sufficiently large number of participants to have a) a training dataset to determine subgroups based on 14 variables and b) a test dataset to determine whether the subgroup solution based on the same

For peer review only

14 variables is similar to the training dataset. This data needs to be collected in both Wave 2 (training dataset) and Wave 3 (test dataset [T1]) combined. Moreover, to test the temporal stability of the subgroups obtained, we require T2 data from different cohorts of participants (Wave 3 and Wave 4). We expect subgroups both within the ASC group, and in a combined sample of ASC + COM. Through simulation, we find that to reliably recover three subgroups, we require at least 160 participants. Therefore, we need to collect data from a minimum of 240 participants at T2 (160 ASC, 80 COM). In addition, we collect a minimum of 30 participants with ADHD at T2 (also see Figure 1 and 2). The attrition rate from Wave 3 to Wave 4 is expected to be lower (=/- 40% across ASC and COM groups) for the questionnaire data given that people do not need to come to the lab. For the ADHD group we estimated the attrition rate to be +/- 50%.

-Insert Figure 2 here-

Ethics and dissemination.

This study is low risk in terms of ethical considerations, since participants will not receive an intervention or rules to live by. Moreover, there is no medical risk associated with participation. Apart from time and energy investment, there is no "burden" associated with participation. We chose to exclude those questions in the interview that were meant to determine whether there are current suicidal thoughts as affirmative answers as such questions could give raise to an ethical dilemma. Moreover, while we do use questionnaires and interviews which are often used in clinical practice during ASC assessment, these instruments cannot be used as standalone diagnostic instruments. Hence, based on these instruments one cannot conclude for clinical purposes whether someone received an unjustified ASC diagnosis or whether they should have an additional diagnosis of a co-

For peer review only

occurring condition. Consequently, we did not communicate about the individual outcomes with participants of these tests in accordance with the rules as set by the local ethical review board. Ethical approval for this study was obtained from the local ethical review board (Wave 1 2011-PN-1952 and 2013-PN-2668, Wave 2 2015-BC-4270, Wave 3 and 4 2018-BC-9285). Local research committees of the participating clinical centers will need to give additional approval for the study. Thus, all procedures performed were in accordance with the ethical standards of the institutional ethical and research committees and with the 1964 Helsinki declaration and its later amendments or comparable ethical standards.

Study results will be published in peer-reviewed (inter)national journals (open access), and presented at conferences. Participants of all waves will receive information regarding the outcomes on a group level and receive a newsletter throughout the duration of the study when they consented to receive such information. Furthermore, these yearly newsletters are also published on our website (dutcharc.nl) and at the end of this overarching project a conference will be organized to which different stakeholders (participants, autistic adults and their relatives, clinicians, and researchers) will be invited. In both 2016 (main focus participants and clinicians of participating clinical centers) and 2018 (all aforementioned groups) such conferences were already successfully organized.

For peer review only

Conclusion

The results of this accelerated longitudinal study will enhance our understanding of the heterogeneity in aging in autistic adults. If the earlier findings regarding protective aging will be replicated longitudinally this will show the strength of neurodiversity which can have positive impact on the (self) stigma regarding ASC. Moreover, the findings will deepen the understanding of the crucial factors influencing the pros and cons of information processing strategies of those with an ASC diagnosis in mid and late adulthood. Information regarding

successful and unsuccessful information processing strategies are useful for autistic individuals, but also for non-autistic individuals, given that this can also inform models about typical aging.

If valid ASC subtypes can be formed, this is relevant for both clinical practice and research. First of all, autistic adults can be informed more specifically about their future outlook (i.e., prognosis), but more importantly in which personal or environmental domains changes can be made in order to increase the likelihood of having a more positive outcome. This is especially relevant when focusing on co-occurring conditions and quality of life as outcome measures, but could also be informative when focusing on cognition. Second, better behaviorally defined subtypes (i.e., endophenotypes) can serve as input for theory forming regarding cause and consequences. Third, if the subtypes are not specific for ASC and thus are equally relevant for ADHD we might need to reconsider the current nosology.

The current study will result in future research question as based on this specific study we will not yet know how the cognitive strategies are different, which interventions (including societal changes) might be a helpful to alter people's future outcomes (like quality of life and/or co-occuring conditions), and how the potential subtypes will develop across a longer time frame. Thus, the current study can serve as input and starting point for future studies and future models regarding aging in autistic adults specifically and neurodevelopmental conditions generally.

For peer review only

Contributorship statement

	HMG	AGL[a]	IG[a]	TR	JAR	CT	WvdP	APG
Funding	■							
Study design Wave 1	■	■						
Study design Wave 2	■		■					
Study design subgrouping (across Waves)	■			■	■			
Study design cognition (across Waves)	■	■				■		
Data collection		■	■	■		■	■	■
First draft of the manuscript	■							■
Feedback on subsequent versions of manuscript	■			■	■	■	■	■
Final approval of the manuscript	■			■	■	■	■	■

[a] was not involved in preparation of the current manuscript, and is thus not one of the authors

Funding statement

This study was funded by the Grant Sponsor is the Netherlands Organization for Scientific Research (NWO); VIDI Grant (grant number: 452–10-003 and VICI Grant (grant number: 453–16-006) awarded to HMG. This funding organization has no involvement in study design, analysis, or interpretation of the data but does require open access publication of the findings.

For peer review only

Data sharing statement

Analyzed data is directly available when requested from the first author during peer review and after publication by non-commercial researchers affiliated with a university. Within five years after the planned future publications, data will become available upon request for non-commercial researchers affiliated with a university to answer additional questions via a repository of the University of Amsterdam.

Competing interest statement

None of the authors have competing interest relevant to the work to report.

Acknowledgements

We want to thank the past, current, and future participants. The students who helped with the data collections as previous, current, and future research assistants. We also thank the institutions and organizations who helped/are helping with recruitment. We are especially grateful by the time and advice provided by the older autistic adults in our think tank.

References

1 American Psychiatric Association. *Diagnostic and Statistical Manual of Mental Disorders*. Fifth Edition. American Psychiatric Association 2013. doi:10.1176/appi.books.9780890425596

2 Brugha TS, McManus S, Smith J, *et al.* Validating two survey methods for identifying cases of autism spectrum disorder among adults in the community. *Psychol Med* 2011;:1–10. doi:S0033291711001292 [pii] 10.1017/S0033291711001292

3 Chiarotti F, Venerosi A. Epidemiology of Autism Spectrum Disorders: A Review of Worldwide Prevalence Estimates Since 2014. *Brain Sci* 2020;**10**:274. doi:10.3390/brainsci10050274

4 Happé F, Frith U. Annual Research Review: Looking back to look forward – changes in the concept of autism and implications for future research. *J Child Psychol Psychiatry* 2020;**61**:218–32. doi:10.1111/jcpp.13176

For peer review only

5 Howlin P, Savage S, Moss P, *et al.* Cognitive and language skills in adults with autism: a 40-year follow-up. *J Child Psychol Psychiatry* 2014;**55**:49–58. doi:10.1111/jcpp.12115

6 Lombardo MV, Lai M-C, Baron-Cohen S. Big data approaches to decomposing heterogeneity across the autism spectrum. *Mol Psychiatry* 2019;**24**:1435–50. doi:10.1038/s41380-018-0321-0

7 Veenstra-VanderWeele J, Blakely RD. Networking in Autism: Leveraging Genetic, Biomarker and Model System Findings in the Search for New Treatments. *Neuropsychopharmacology* 2012;**37**:196–212. doi:10.1038/npp.2011.185

8 Mukaetova-Ladinska EB, Perry E, Baron M, *et al.* Ageing in people with autistic spectrum disorder: Ageing in people with autistic spectrum disorder. *Int J Geriatr Psychiatry* 2012;**27**:109–18. doi:10.1002/gps.2711

9 Roestorf A, Bowler DM, Deserno MK, *et al.* "Older Adults with ASD: The Consequences of Aging." Insights from a series of special interest group meetings held at the International Society for Autism Research 2016–2017. *Res Autism Spectr Disord* 2019;**63**:3–12. doi:10.1016/j.rasd.2018.08.007

10 Geurts HM, Vissers ME. Elderly with autism: executive functions and memory. *J Autism Dev Disord* 2012;**42**:665–75. doi:10.1007/s10803-011-1291-0

11 Happé F, Charlton RA. Aging in autism spectrum disorders: a mini-review. *Gerontology* 2012;**58**:70–8. doi:10.1159/000329720

12 Lever AG, Werkle-Bergner M, Brandmaier AM, *et al.* Atypical working memory decline across the adult lifespan in autism spectrum disorder? *J Abnorm Psychol* 2015;**124**:1014–26. doi:10.1037/abn0000108

13 Lever AG, Ridderinkhof KR, Marsman M, *et al.* Reactive and proactive interference control in adults with autism spectrum disorder across the lifespan. *Dev Psychol* 2017;**53**:379–95. doi:10.1037/dev0000219

14 Lever AG, Geurts HM. Age-related differences in cognition across the adult lifespan in autism spectrum disorder. *Autism Res Off J Int Soc Autism Res* 2016;**9**:666–76. doi:10.1002/aur.1545

15 Demetriou EA, Lampit A, Quintana DS, *et al.* Autism spectrum disorders: a meta-analysis of executive function. *Mol Psychiatry* 2018;**23**:1198–204. doi:10.1038/mp.2017.75

16 Davids RCD, Groen Y, Berg IJ, *et al.* Executive Functions in Older Adults With Autism Spectrum Disorder: Objective Performance and Subjective Complaints. *J Autism Dev Disord* 2016;**46**:2859–73. doi:10.1007/s10803-016-2831-4

17 Geurts HM, Pol SE, Lobbestael J, *et al.* Executive Functioning in 60+ Autistic Males: The Discrepancy Between Experienced Challenges and Cognitive Performance. *J Autism Dev Disord* Published Online First: 17 January 2020. doi:10.1007/s10803-020-04368-9

For peer review only

18 Braden BB, Smith CJ, Thompson A, *et al.* Executive function and functional and structural brain differences in middle-age adults with autism spectrum disorder. *Autism Res Off J Int Soc Autism Res* 2017;**10**:1945–59. doi:10.1002/aur.1842

19 Powell PS, Klinger LG, Klinger MR. Patterns of Age-Related Cognitive Differences in Adults with Autism Spectrum Disorder. *J Autism Dev Disord* 2017;**47**:3204–19. doi:10.1007/s10803-017-3238-6

20 Tse VWS, Crabtree J, Islam S, *et al.* Comparing Intellectual and Memory Abilities of Older Autistic Adults with Typically Developing Older Adults Using WAIS-IV and WMS-IV. *J Autism Dev Disord* 2019;**49**:4123–33. doi:10.1007/s10803-019-04122-w

21 Walsh MJM, Baxter LC, Smith CJ, *et al.* Age group differences in executive network functional connectivity and relationships with social behavior in men with autism spectrum disorder. *Res Autism Spectr Disord* 2019;**63**:63–77. doi:10.1016/j.rasd.2019.02.008

22 McPartland JC. Considerations in biomarker development for neurodevelopmental disorders: *Curr Opin Neurol* 2016;**29**:118–22. doi:10.1097/WCO.0000000000000300

23 Cholemkery H, Medda J, Lempp T, *et al.* Classifying Autism Spectrum Disorders by ADI-R: Subtypes or Severity Gradient? *J Autism Dev Disord* 2016;**46**:2327–39. doi:10.1007/s10803-016-2760-2

24 Rommelse NNJ, Geurts HM, Franke B, *et al.* A review on cognitive and brain endophenotypes that may be common in autism spectrum disorder and attention-deficit/hyperactivity disorder and facilitate the search for pleiotropic genes. *Neurosci Amp Biobehav Rev* 2011;**35**:1363–96.

25 Bathelt J, Koolschijn PC, Geurts HM. Age-variant and age-invariant features of functional brain organization in middle-aged and older autistic adults. *Mol Autism* 2020;**11**:9. doi:10.1186/s13229-020-0316-y

26 Koolschijn PCMP, Caan MWA, Teeuw J, *et al.* Age-related differences in autism: The case of white matter microstructure. *Hum Brain Mapp* 2017;**38**:82–96. doi:10.1002/hbm.23345

27 Koolschijn PCMP, Geurts HM. Gray Matter Characteristics in Mid and Old Aged Adults with ASD. *J Autism Dev Disord* 2016;**46**:2666–78. doi:10.1007/s10803-016-2810-9

28 Wechsler. WAIS-III. *San Antonio Psychol Corp* 1997.

29 Wechsler. WAIS-IV. *San Antonio Psychol Corp* 2008.

30 Uterwijk J. WAIS-III Nederlandstalige bewerking. Technische handleiding. 2000.

31 American Psychiatric Association. *Diagnostic and Statistical Manual of Mental Health Disorders*. Washington DC: American Psychiatric Association 1994.

32 Baron-Cohen, Wheelwright S, Skinner R, *et al.* The autism-spectrum quotient (AQ): evidence from Asperger syndrome/high-functioning autism, males and females, scientists and mathematicians. *J Autism Dev Disord* 2001;**31**:5–17. doi:10.1023/a:1005653411471

For peer review only

33 Kooij JS, Buitelaar JK, FURER JW, *et al.* Internal and external validity of attention-deficit hyperactivity disorder in a population-based sample of adults. *Psychol Med* 2005;**35**:817–827.

34 Folstein MF, Folstein SE, McHugh PR. "Mini-mental state": a practical method for grading the cognitive state of patients for the clinician. *J Psychiatr Res* 1975;**12**:189–98.

35 Hoekstra RA, Bartels M, Cath DC, *et al.* Factor structure, reliability and criterion validity of the Autism-Spectrum Quotient (AQ): a study in Dutch population and patient groups. *J Autism Dev Disord* 2008;**38**:1555–66. doi:10.1007/s10803-008-0538-x

36 Davis MH. A multidimensional approach to individual differences in empathy. 1980.

37 De Corte K, Buysse A, Verhofstadt LL, *et al.* Measuring empathic tendencies: Reliability and validity of the Dutch version of the Interpersonal Reactivity Index. *Psychol Belg* 2007;**47**:235–260.

38 Minshew NJ, Hobson JA. Sensory sensitivities and performance on sensory perceptual tasks in high-functioning individuals with autism. *J Autism Dev Disord* 2008;**38**:1485–98.

39 Arrindell W, Ettema J. Symptom checklist: Handleiding bij een multidimensionale psychopathologie-indicator SCL-90. 2005.

40 Derogatis LR. Symptoms checklist-90. *Adm Scoring Proced Man Revis Version* 1977.

41 Central Bureau of Statistics (CBS). Health Interview Questionnaire. *Heerlen Neth* 1989.

42 Craig CL, Marshall AL, Sjöström M, *et al.* International physical activity questionnaire: 12-country reliability and validity. *Med Sci Sports Exerc* 2003;**35**:1381–1395.

43 Bartels M, Boomsma DI. Born to be Happy? The Etiology of Subjective Well-Being. *Behav Genet* 2009;**39**:605–15. doi:10.1007/s10519-009-9294-8

44 WHOQoL Group. Development of the World Health Organization WHOQOL-BREF quality of life assessment. *Psychol Med* 1998;**28**:551–558.

45 Trompenaars FJ, Masthoff ED, Van Heck GL, *et al.* Content validity, construct validity, and reliability of the WHOQOL-Bref in a population of Dutch adult psychiatric outpatients. *Qual Life Res* 2005;**14**:151–160.

46 Marks IM, Mathews AM. Brief standard self-rating for phobic patients. *Behav Res Ther* 1979;**17**:263–267.

47 Wisocki PA, Handen B, Morse CK. The Worry Scale as a measure of anxiety among homebound and community active elderly. *Behav Ther* 1986.

48 Pearlin LI, Schooler C. The structure of coping. *J Health Soc Behav* 1978;:2–21.

49 Broadbent DE, Cooper PF, FitzGerald P, *et al.* The cognitive failures questionnaire (CFQ) and its correlates. *Br J Clin Psychol* 1982;**21**:1–16.

For peer review only

50 Merckelbach H, Muris P, Nijman H, *et al.* Self-reported cognitive failures and neurotic symptomatology. *Personal Individ Differ* 1996;**20**:715–724.

51 Hull L, Mandy W, Lai M-C, *et al.* Development and validation of the camouflaging autistic traits questionnaire (CAT-Q). *J Autism Dev Disord* 2019;**49**:819–33.

52 Brugha TS, McManus S, Bankart J, *et al.* Epidemiology of autism spectrum disorders in adults in the community in England. *Arch Gen Psychiatry* 2011;**68**:459–465.

53 Peeters F, Ponds R, Vermeeren MTG. Affectiviteit en zeltbeoordeling van depressie en angst. *Tijdschr Voor Psychiatr* 1996;**38**:240–250.

54 Watson D, Clark LA, Tellegen A. Development and validation of brief measures of positive and negative affect: the PANAS scales. *J Pers Soc Psychol* 1988;**54**:1063.

55 Stansfeld S, Marmot M. Deriving a survey measure of social support: the reliability and validity of the Close Persons Questionnaire. *Soc Sci Med* 1992;**35**:1027–1035.

56 Agelink van Rentergem J, Radhoe T, Geurts HM. A Dutch translation of the CAT-Q.

57 Hull L, Mandy W, Lai M-C, *et al.* Development and validation of the camouflaging autistic traits questionnaire (CAT-Q). *J Autism Dev Disord* 2019;**49**:819–833.

58 Greene JG. Measuring the symptom dimension of quality of life: General and menopause-specific scales and their subscale structure. *Horm Replace Ther Qual Life* 2002.

59 Kok R, Verhey F. Dutch translation of the mini mental state examination (Folstein et al., 1975). *Altrecht GGZ* 2002.

60 Bildt A, Greaves-Lord K, De Jonge M. ADOS-2 Nederlandse bewerking.[ADOS-2 Dutch Adaptation]. *Amst Neth Hogrefe Uitgevers* 2013.

61 Lord C, Risi S, Lambrecht L, *et al.* The Autism Diagnostic Observation Schedule—Generic: A standard measure of social and communication deficits associated with the spectrum of autism. *J Autism Dev Disord* 2000;**30**:205–223.

62 Vuijk R. *Nederlands Interview ten behoeve van Diagnostiek Autismespectrumstoornis bij Volwassenen (NIDA)*. Amsterdam: Boom Uitgevers 2014.

63 Sheehan DV, Lecrubier Y, Sheehan KH, *et al.* The validity of the Mini International Neuropsychiatric Interview (MINI) according to the SCID-P and its reliability. *Eur Psychiatry* 1997;**12**:232–241.

64 Van Vliet I, Leroy H, Van Megen H. De MINI internationaal neuropsychiatrisch interview, nederlandse versie 5.0. 0. *Leiden Leiden Univ Med Cent Dep Psychiatry* 2000.

65 Rey A. L'examen psychologique dans les cas d'encéphalopathie traumatique.(Les problems.). *Arch Psychol* 1941.

66 Wechsler D. Wechsler memory scale (WMS-III)(Vol. 14). *San Antonio TX Psychol Corp* 1997.

For peer review only

67 Altgassen M, Koban N, Kliegel M. Do Adults with Autism Spectrum Disorders Compensate in Naturalistic Prospective Memory Tasks? *J Autism Dev Disord* 2012;**42**:2141–51. doi:10.1007/s10803-012-1466-3

68 Craik FI, Bialystok E. Planning and task management in older adults: Cooking breakfast. *Mem Cognit* 2006;**34**:1236–49.

69 Spek AA, Scholte EM, Van Berckelaer-Onnes IA. Theory of Mind in Adults with HFA and Asperger Syndrome. *J Autism Dev Disord* 2010;**40**:280–9. doi:10.1007/s10803-009-0860-y

70 Stone VE, Baron-Cohen S, Knight RT. Frontal Lobe Contributions to Theory of Mind. *J Cogn Neurosci* 1998;**10**:640–56. doi:10.1162/089892998562942

71 Luteijn F, Barelds DPH. *GIT2: Groninger Intelligentie Test 2*. Harcourt Test Publishers 2004.

72 Shallice T. Specific impairments of planning. *Philos Trans R Soc Lond B Biol Sci* 1982;**298**:199–209.

73 Reitan RM, Wolfson D. TheHalslead-Reitan Neuropsychological Jesi Battery: theory and interpretai ion. 1985.

74 Lever AG, Geurts HM. Psychiatric Co-occurring Symptoms and Disorders in Young, Middle-Aged, and Older Adults with Autism Spectrum Disorder. *J Autism Dev Disord* 2016;**46**:1916–30. doi:10.1007/s10803-016-2722-8

75 Lever AG, Geurts HM. Is Older Age Associated with Higher Self- and Other-Rated ASD Characteristics? *J Autism Dev Disord* 2018;**48**:2038–51. doi:10.1007/s10803-017-3444-2

76 Fabbri E, An Y, Zoli M, *et al.* Association Between Accelerated Multimorbidity and Age-Related Cognitive Decline in Older Baltimore Longitudinal Study of Aging Participants without Dementia. *J Am Geriatr Soc* 2016;**64**:965–72. doi:10.1111/jgs.14092

77 Moerbeek M. The Effects of the Number of Cohorts, Degree of Overlap Among Cohorts, and Frequency of Observation on Power in Accelerated Longitudinal Designs. *Methodology* 2011;**7**:11–24. doi:10.1027/1614-2241/a000019

For peer review only

Tables and Figures

Table 1. Timing and use of questionnaires

Measures		Waves				Study focus	
Instrument	Construct	1	2	3	4	Aim 1	Aim 2
General	Descriptives					INCL	INCL/SUB
AQ- self	Autism traits					INCL	SUB
AQ- proxy	Autism traits						
IRI-self	Personal relations						
IRI-proxy	Personal relations						
SSQ-self	Sensory sensitivities						SUB
SSQ-proxy	Sensory sensitivities						
ADHD-SR	ADHD traits					INCL	
SCL-90	Mental health						VAL
Health Ques	Physical health					INCL	
MRS	Menopause						
CFQ	Global cognition					REP	VAL
PANAS	Global emotion						SUB
WHOQoL	Quality of Life						VAL
Happiness	Well-being						
Mastery	Stress/Self-control						SUB
Worry	Stress/Worries						SUB
IPAQ	Physical activity						SUB
Brugha	Life events						SUB
CPI	Social network						SUB
CAT-Q	Camouflaging						

For peer review only

Note. Aim 1: Arbitrate between cognitive aging hypotheses; Aim 2: Subgrouping; Gray shading indicates that a measure was administered during that Wave. ADHD-SR=Attention Deficit Hyperactivity Disorder Self Report; AQ=Autism spectrum Quotient*;* CFQ=Cognitive Failure Questionnaire; Brugha=Brugha questionnaire of important life events during childhood and in the past years; CPQ=Close Persons Questionnaire to measure present social support from the four most intimate persons; INCL= used for inclusion; IPAQ=International Physical Activities Questionnaire; Happiness: Perceived well-being question of the NAR; IRI= interpersonal reactivity inventory; MRS= menopause rating scale; PANAS=Positive and Negative Affect Schedule; REP= Replication; SCL-90=Symptom Checklist; SSQ=Sensory Sensitivity Questionnaire; SUB= used for subtyping; VAL= Validation; WHOQoL=World Health Organization Quality of Life.

Table 2. Timing and use of diagnostic measures

Diagnostic assessment								
	Measures		Waves				Study focus	
Type	Instrument	Construct	1	2	3	4	Aim 1	Aim 2
CNP	MMSE	Global cognition					INCL	
CNP	WAIS subtests	Estimation of intelligence					INCL	INCL
OBS	ADOS	ASC					INCL	
INT	NIDA	ASC						
INT	MINI	Mental health					INCL	VAL

Note. Gray shading indicates that a measure was administered during that Wave. ADOS= Autism Diagnostic Observation Scale; CNP= clinical neuropsychologic task; INCL= used for inclusion; INT= Interview; MINI= MINI the International Neuropsychiatric Interview; MMSE=Mini Mental State Examination; NIDA= Nederlands Interview ten behoeve van Diagnostiek Autismespectrumstoornis bij volwassenen [Dutch Interview for ASD assessment in adults]; OBS = Observation; VAL: Measure to test validity of subtypes.

Table 3. Timing and use of cognitive measures

Cognitive assessment						
	Measures		Waves			
Type	Instrument	Construct	1	2	3	4
CNP	RAVLT	Episodic verbal memory				
CNP	WMS	Episodic visual memory				
CNP	DAT & COWAT	EF (generativity)				
CNP	TMT A & B	EF (flexibility)				
CNP	ToL	EF (planning)				
EXP	Go-NoGo	EF (inhibition)				
EXP	Simon	EF (inhibition)				
EXP	N-back	EF (working memory)				
EXP	2-Choice RT	Processing speed				
CNP	Faux Pas	Social cognition (ToM)				
EXP	Odd ball	Attention				
EXP	ABT	Prospective memory				

Note. Gray shading indicates that a measure was administered during that Wave. ABT= Amsterdam Breakfast Task; CNP= clinical neuropsychologic task; DAT=Dutch version of the Controlled Word Association Test; EF=Executive functioning; EXP= experimental task; RAVLT=Rey Auditory Verbal Learning Test; 2-Choice RT=a simple computerized reaction time task; Simon=a computerized inhibition task; ToL= Tower of London; ToM=Theory of Mind; WMS-III=Wechsler Memory Scale 3rd edition, subtest visual reproduction.

Figure captions

Figure 1. Flow charts for each separate cohort.

Note. Cohort 1 (left panel) started in March 2012, Cohort 2 (middle panel) started in December 2015. Cohort 3 started in September 2018. Wave 3 is currently being collected. Data collection on Wave 4 is estimated to start end 2020, numbers reported for Wave 4 are based on expected attrition rates (i.e., 40% for questionnaires, for cognition 30% for ASC, 50% for comparisons). Participant numbers in Wave 3 are based on current inclusion in combination with our power analyses for Wave 4. From Wave 1 to Wave 2 the inclusion age increased from 20 to 30 years old (see main text). = indicates exact numbers; ≥ indicates planned numbers; ≈ estimated numbers based on expected attrition rates. ADHD: Attention-deficit/ hyperactivity disorder; ASC: Autism spectrum condition; C: Cognitive tests; COM: comparison; Q: Questionnaires

Figure 2. Total number of participants seen (Wave 1 and 2) or need to be seen minimally (Wave 3 and 4) at each wave when taking into account the estimated attrition rate (see attrition) and age inclusion criterion (>30 years) at time of inclusion (see text).

Note. = indicates exact numbers; ≥ planned numbers; ≈ estimated numbers based on expected attrition rates. ADHD: Attention-deficit/ hyperactivity disorder; ASC: Autism spectrum condition; C: Cognitive tests; COM: comparison; Q: Questionnaires. N.B. reported numbers at Wave 4 are based on power analyses. The number of newly recruited participants can be seen in Figure 1.

For peer review only

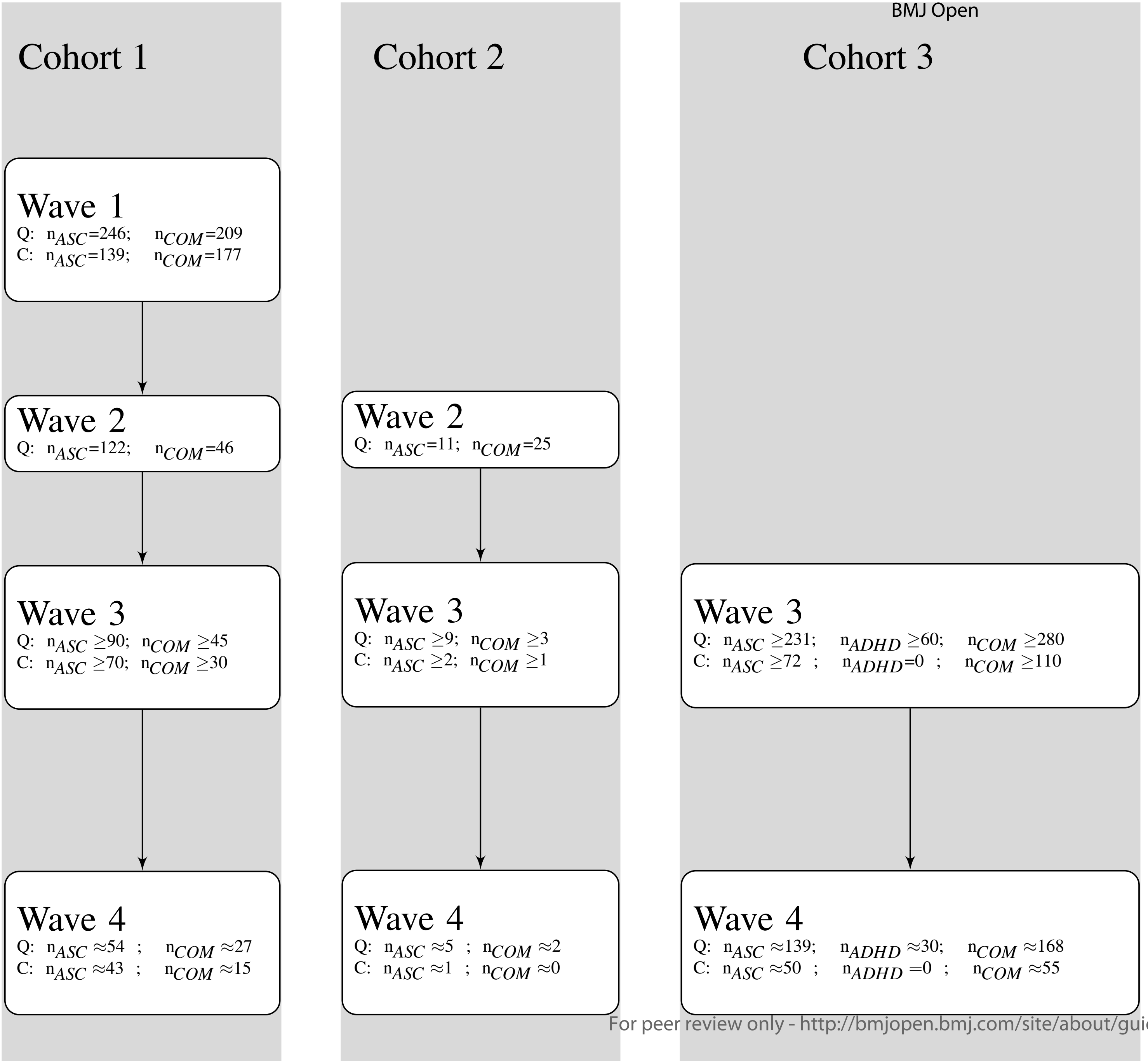
Cohort 1
Cohort 2
Cohort 3
Wave 1
Q: n_{ASC}=246; n_{COM}=209
C: n_{ASC}=139; n_{COM}=177
Wave 2
Q: n_{ASC}=122; n_{COM}=46
Wave 2
Q: n_{ASC}=11; n_{COM}=25
Wave 3
Q: $n_{ASC} \geq$90; $n_{COM} \geq$45
C: $n_{ASC} \geq$70; $n_{COM} \geq$30
Wave 3
Q: $n_{ASC} \geq$9; $n_{COM} \geq$3
C: $n_{ASC} \geq$2; $n_{COM} \geq$1
Wave 3
Q: $n_{ASC} \geq$231; $n_{ADHD} \geq$60; $n_{COM} \geq$280
C: $n_{ASC} \geq$72 ; n_{ADHD}=0 ; $n_{COM} \geq$110
Wave 4
Q: $n_{ASC} \approx$54 ; $n_{COM} \approx$27
C: $n_{ASC} \approx$43 ; $n_{COM} \approx$15
Wave 4
Q: $n_{ASC} \approx$5 ; $n_{COM} \approx$2
C: $n_{ASC} \approx$1 ; $n_{COM} \approx$0
Wave 4
Q: $n_{ASC} \approx$139; $n_{ADHD} \approx$30; $n_{COM} \approx$168
C: $n_{ASC} \approx$50 ; n_{ADHD} =0 ; $n_{COM} \approx$55

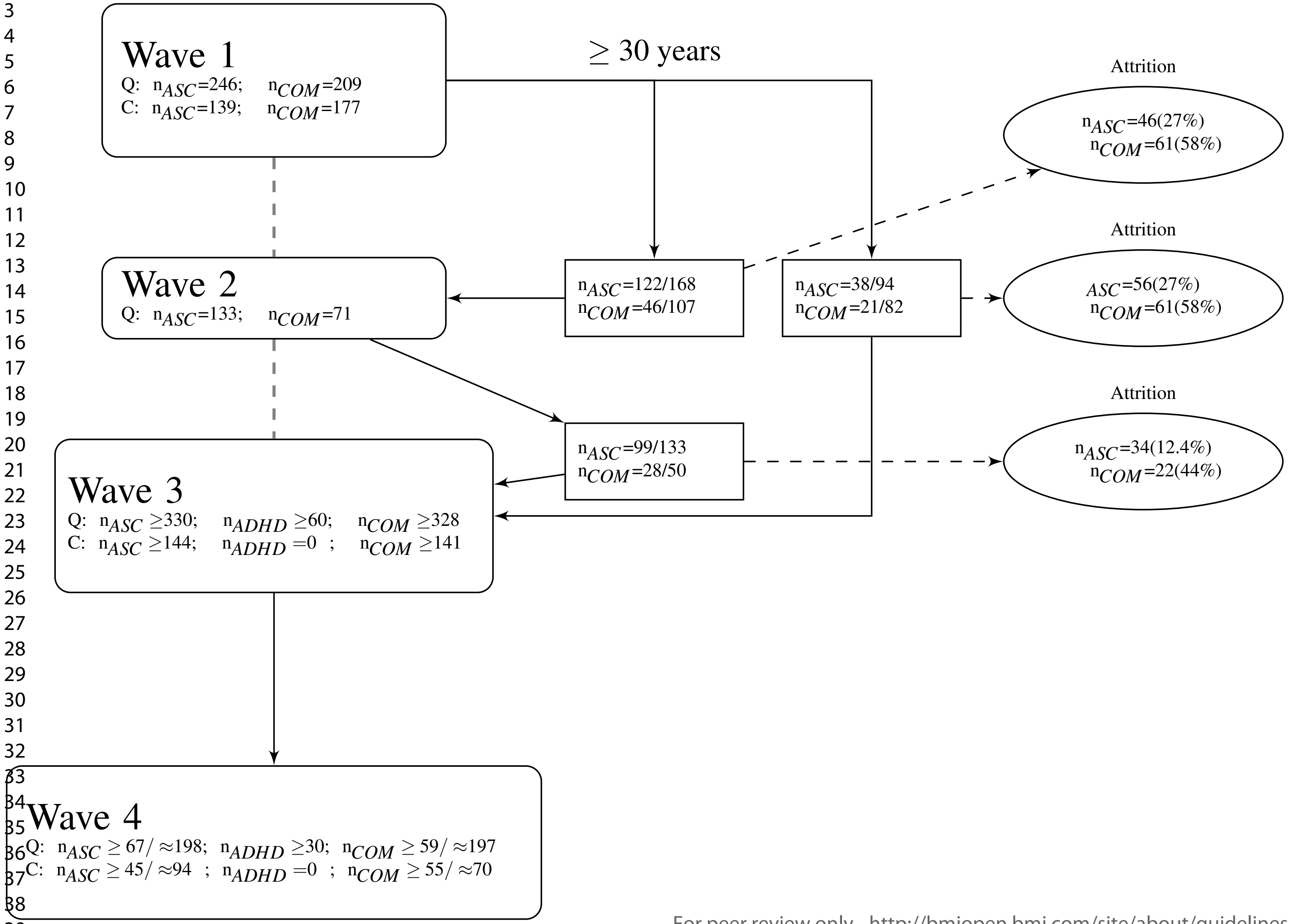
Wave 1
Q: nASC=246; nCOM=209
C: nASC=139; nCOM=177
≥ 30 years
Attrition
nASC=46(27%)
nCOM=61(58%)
Attrition
ASC=56(27%)
nCOM=61(58%)
Attrition
nASC=34(12.4%)
nCOM=22(44%)
Wave 2
Q: nASC=133; nCOM=71
nASC=122/168
nCOM=46/107
nASC=38/94
nCOM=21/82
nASC=99/133
nCOM=28/50
Wave 3
Q: nASC ≥330; nADHD ≥60; nCOM ≥328
C: nASC ≥144; nADHD =0 ; nCOM ≥141
Wave 4
Q: nASC ≥ 67/ ≈198; nADHD ≥30; nCOM ≥ 59/ ≈197
C: nASC ≥ 45/ ≈94 ; nADHD =0 ; nCOM ≥ 55/ ≈70

BMJ Open

Aging and heterogeneity regarding autism spectrum conditions: A protocol paper of an accelerated longitudinal study

Journal:	*BMJ Open*
Manuscript ID	bmjopen-2020-040943.R1
Article Type:	Protocol
Date Submitted by the Author:	21-Dec-2020
Complete List of Authors:	Geurts, Hilde M.; University of Amsterdam, Psychology; Dr Leo Kannerhuis (Youz),<br>Agelink van Rentergem, Joost; University of Amsterdam, Psychology<br>Radhoe, Tulsi; University of Amsterdam, Psychology<br>Torenvliet, Carolien; University of Amsterdam, Psychology<br>Van der Putten, Wikke J; University of Amsterdam, Psychology; Dr Leo Kannerhuis (Youz),<br>Groenman, Annabeth P; University of Amsterdam, Psychology
**Primary Subject Heading**:	Mental health
Secondary Subject Heading:	Geriatric medicine
Keywords:	Adult psychiatry < PSYCHIATRY, Old age psychiatry < PSYCHIATRY, Developmental neurology & neurodisability < PAEDIATRICS

SCHOLARONE™
Manuscripts

I, the Submitting Author has the right to grant and does grant on behalf of all authors of the Work (as defined in the below author licence), an exclusive licence and/or a non-exclusive licence for contributions from authors who are: i) UK Crown employees; ii) where BMJ has agreed a CC-BY licence shall apply, and/or iii) in accordance with the terms applicable for US Federal Government officers or employees acting as part of their official duties; on a worldwide, perpetual, irrevocable, royalty-free basis to BMJ Publishing Group Ltd ("BMJ") its licensees and where the relevant Journal is co-owned by BMJ to the co-owners of the Journal, to publish the Work in this journal and any other BMJ products and to exploit all rights, as set out in our licence.

The Submitting Author accepts and understands that any supply made under these terms is made by BMJ to the Submitting Author unless you are acting as an employee on behalf of your employer or a postgraduate student of an affiliated institution which is paying any applicable article publishing charge ("APC") for Open Access articles. Where the Submitting Author wishes to make the Work available on an Open Access basis (and intends to pay the relevant APC), the terms of reuse of such Open Access shall be governed by a Creative Commons licence – details of these licences and which Creative Commons licence will apply to this Work are set out in our licence referred to above.

Other than as permitted in any relevant BMJ Author's Self Archiving Policies, I confirm this Work has not been accepted for publication elsewhere, is not being considered for publication elsewhere and does not duplicate material already published. I confirm all authors consent to publication of this Work and authorise the granting of this licence.

For peer review only

Aging and heterogeneity regarding autism spectrum conditions:

A protocol paper of an accelerated longitudinal study

Geurts, H.M.[1,2] , Agelink van Rentergem, J.[1],

Radhoe, T.[1], Torenvliet, C. [1], Van der Putten, W.J.[1,2] , Groenman, A.P.[1]

[1]Dutch Autism & ADHD Research Center, Department of Psychology, University of Amsterdam, Amsterdam, the Netherlands

[2]Dr. Leo Kannerhuis, autism clinic (Youz/Parnassia Group), Amsterdam, the Netherlands

Address correspondence to:

Correspondence author information: Hilde M. Geurts, H.M.Geurts@uva.nl, phone +31 20 525 6843, Dutch Autism and ADHD Research Center (d'Arc), Brain & Cognition, Department of Psychology, University of Amsterdam, Postbus 15915, 1001 NK, Amsterdam, The Netherlands.

Number of words (From Introduction till References & Tables): 4952

Number of Tables: 4

Number of Figures: 2

Appendix: 1

For peer review only

Abstract

Introduction: Autism spectrum conditions (ASC) develop early in life and are thought to last a lifetime. However, ASC research has two major knowledge gaps that hinder progression in understanding the concept of ASC and in providing proper support for autistic adults: 1) the majority of knowledge about ASC mainly stems from childhood studies so little is known about older autistic adults; 2) while it is broadly recognized that ASC is a heterogeneous condition, we do not yet understand the differences in trajectories leading to their future outcome. We aim to fill both knowledge gaps.

Methods and analysis: A multistage overlapping cohorts design assessing (cognitive) aging in ASC, is designed to obtain an accelerated longitudinal dataset. Data, including a multitude of questionnaires, diagnostics, and cognitive tests, are collected over four waves within a 10-year time frame. This will provide information regarding actual changes in quality of life, co-occurring health conditions, and cognition as well as the possibility to test external validity and temporal stability in newly formed behavioral subtypes. Participants consist of three groups of adults aged 20 to 90 years: 1) with a clinical diagnosis of ASC; 2) with a clinical diagnosis of ADHD, but no ASC; 3) no ASC/ADHD (i.e., comparison group; COM). The sample size differs between waves and instruments. Detailed analysis plans will be preregistered in AsPredicted or at the Open Science Framework.

Ethics and dissemination: Ethical approval for this study was obtained from the ethical review board of the Department of Psychology of the University of Amsterdam (Wave 1 2011-PN-1952 and 2013-PN-2668, Wave 2 2015-BC-4270, Wave 3 and 4 2018-BC-9285). In line with the funding policies of the grant organization funding this study, future papers will be published open access.

KEY WORDS: Autism, Aging, Heterogeneity, Cognition, Protocol, Longitudinal

For peer review only

Strengths and limitations of this study

Strengths are:

1. This study has an accelerated longitudinal design, so in a relative short time frame for the first time actual age-related (cognitive) changes can be assessed in older autistic adults.
2. We will use in-depth phenotyping due to inclusion of measures of known vulnerability and protective factors regarding aging and a wide range of diagnostic instruments, questionnaires, and cognitive tests.
3. The replicability, external validity, and temporal stability of behavioral ASC subgroups can be tested, which are aspects that are often not implemented in subgroup study designs.

Limitations are:

1. The first two measurement waves were not set up as being part of an accelerated longitudinal design which led to high attrition rates.
2. As such, not all measures are the same across all four measurement waves.

For peer review only

Introduction

Neurodevelopmental conditions, like an autism spectrum condition (ASC), are defined as conditions with early atypical brain development, which have cascading effects on cognitive processes important during the entire lifespan [1]. At the core of the Diagnostic and Statistical Manual of Mental Health Disorders Fifth Edition (DSM-5) ASC diagnosis are difficulties in social interaction and communication, sensory sensitivities, and stereotyped or repetitive behaviors and interests [1]. A conservative estimate is that one in hundred persons meets the criteria [2], but recent estimates seem to be higher and vary widely [3]. Whereas ASC was originally perceived as a childhood condition, which was often comorbid with a severe intellectual disability and had an overall poor outcome, we now know that ASCs are not restricted to childhood or to those with low intellectual functioning, and that there is large heterogeneity across persons and across time in type and severity of the disabilities experienced [4,5]. This led to the idea that while ASC is a behaviorally defined condition, it likely consists of different conditions with diverse outcomes which are potentially falsely lumped together in one diagnostic category [6,7]. Understanding the concept, course, and consequences of ASCs is critical in order to develop appropriate support for (old) autistic[1] adults and for adults with related neurodevelopmental conditions. Unfortunately, ASC research hardly exceeded young adulthood, so what happens in old age remained exempt of empirical scrutiny [8,9] (for an overview of reasons for the lack of aging research see [10,11]). Thus, two questions need to be answered:

1. How do people with ASCs progress in late adulthood?
2. How can we understand the differences among people with ASC in future outcome?

[1] We chose to use the term autism spectrum conditions as the traditional and official DSM-5 term "autism spectrum disorder" and we use identity first language as this is what a majority of autistic adults seem to prefer in the UK. We are well aware that in other countries, like the Netherlands, the preference of autistic adults is not to use identity first language, but given that this paper is written in English we chose the UK preference.

For peer review only

While the process of cognitive decline might be similar for autistic adults and non-autistic adults (i.e., parallel aging or delayed aging where cognitive maturation and cognitive decline are shifted in time, but follow a similar pattern), a dominant assumption is that autistic adults are at a larger risk for accelerated cognitive aging [10,12]. However, recent findings from cross-sectional studies suggest that this assumption is neither appropriate for all cognitive domains nor appropriate for all autistic adults. We, for example, found that while autistic adults reported that they are experiencing a large number of cognitive challenges in daily life, these autistic adults hardly differed from controls on a wide range of cognitive domains (e.g., episodic verbal memory, inhibition [13–15]) and not all adults experience the same cognitive challenges [15]. The ASC group performed worse with respect to advanced social knowledge (i.e., mentalizing) and executive functioning (especially the ability to come up with various adequate solutions when confronted with a challenge, i.e., generativity [15]). But, interestingly, the observed effect sizes were small and only a small proportion of the autistic adults seem to have clinically relevant cognitive problems. In studies including autistic children, typically medium effect sizes are observed and performance differences are apparent across a broader range of cognitive domains [16]. Moreover, independent empirical studies including old autistic individuals recently reported similar findings as Lever and colleagues observed [17,18]. So, in sharp contrast to the dominant hypothesis, these novel findings suggest that with increasing age, the cognitive differences between autistic adults and non-autistic adults become less pronounced. Yet, even more challenging for the dominant view, is that strong age-related cognitive differences are found in non-autistic adults, with respect to episodic visual memory and visual working memory, but *not* in autistic adults [13,15]. Also, while across different ages, autistic adults seemed to perform worse with respect to mentalizing, performances of adults with and without an ASC diagnosis did not differ in those over 50 years [15]. Hence, recent findings concerning cognition across adulthood are not just in contrast with the accelerated aging hypothesis, but could even suggest that autistic adults are less sensitive to the typical

For peer review only

cognitive aging effects. This is why we proposed an alternative hypothesis, which is that the cognitive strategies (a subgroup of) autistic adults use, while disadvantageous when young, are beneficial (i.e., protective) when older [12,15]. However, in order to test such a hypothesis, we first need to determine whether previous cross-sectional findings can be replicated cross-sectionally as well as longitudinally, in sufficiently large groups. This is especially of importance as recently various small studies have been published with rather mixed findings regarding which cognitive functions are predominantly associated with specific cognitive aging patterns [18–22].

Moreover, we need to determine whether we can actually form well-validated adult ASC subtypes. If there are indeed adulthood subtypes, various aging hypotheses (i.e. accelerated cognitive decline, protective cognitive strategy use, and delayed aging) can be true for different subtypes in different cognitive domains. Ideally, when defining subtypes, one uses well-accepted low-cost, nonintrusive measures such as short questionnaires [23]. Behaviorally defined subgroups, so far, differed mainly in severity (i.e., quantitative instead of qualitative differences; [24]). However, it could well be that valid subtypes can be formed when symptom information is combined with other important behavioral characteristics, which are in the general population considered to be vulnerability (e.g., stressful life events and lack of control) and protective factors (e.g., physically activity and a sound social network) for a wide range of outcome measures.

These adulthood subtypes are not just of relevance for cognitive aging, but are also of relevance to gain more tailored information regarding someone's prognosis. Such newly formed subtypes need to be tested by checking multiple external validators, such as temporal stability and clinical outcome (next to change in cognitive abilities also the presence of comorbidities, subjective wellbeing) at follow up. Ideally, in order to test the specificity of newly formed subtypes, another clinical group needs to be included. Put differently, including data from an additional neurodevelopmental condition (like ADHD) ensures that we can test

For peer review only

whether the obtained subgroup findings will be specific for ASCs and follow the distinction between ASC and other neurodevelopmental conditions or whether the obtained subtypes cross the classical borders between these conditions. Given the overlap between ASC and ADHD [25], the second scenario is more likely. Hence, the proposed study will not just gain relevant knowledge for those with an ASC diagnosis, but will also add to the understanding of aging in related neurodevelopmental conditions.

In sum, we have two major aims;

1. To arbitrate between contrasting ASC-related cognitive aging hypotheses by studying aging trajectories;
2. To determine (late) adulthood ASC subtypes and to explore if there are specific prognostic behavioral markers that are related to the observed heterogeneity, and can be used in clinical practice to predict who might or might not be prone to a) co-occurring mental health conditions; b) lower quality of life; and c) cognitive decline.

We hypothesize that on overall group level we will replicate the findings of our cross-sectional study [13,15], cross-sectionally as well as longitudinally. This implies that for the majority of cognitive domains, the aging trajectory of autistic adults will parallel the aging trajectory of the controls, but for visual memory, visual working memory, and Theory of Mind the aging pattern would be in line with the protective cognitive strategy hypothesis.

Moreover, we hypothesize that based on a subset of known vulnerability and protective factors for typical accelerated (cognitive) aging next to ASC symptom measures, valid ASC adulthood outcome subtypes can be determined. However, currently there are no longitudinal datasets focusing on autistic adults which include a) a significant proportion of adults over 55 years of age, with b) sufficiently detailed measures on (mental health), quality of life, and objective and subjective cognition; and with c) comparison groups with and

For peer review only

without other neurodevelopmental conditions (i.e., ADHD). Each of the criteria needs to be met in order to properly test the aforementioned hypotheses. Moreover, most earlier studies did not report whether they consulted autistic adults in the research process while the potential outcomes of these study are especially relevant to them. Therefore, we designed the current accelerated longitudinal study with regular input from autistic adults.

Thus, in this study we will map changes across time in cognition, mental health, and subjective well-being in autistic adults and non-autistic adults in mid and late adulthood (30 to 90 years). This way we can test whether we can replicate earlier cognitive findings and we can test specificity, external validity, temporal stability, predictive validity, and replicability for behaviorally defined subtypes in autistic adults.

Methods

Study design

This study follows a multistage overlapping cohorts design assessing (cognitive) aging in ASC including an ASC group, a typically developing comparison group (COM), and an ADHD group. This study was designed to obtain an accelerated longitudinal dataset. Within in each of these three cohorts we start measuring at a different age point, but the cohorts do overlap in the older age ranges. Because of this, we will not have to follow each individual for a full lifetime, to be able to say something about development during adulthood. Essential is that within a relatively short time-frame [26], cognitive alterations can be detected from the age of 30 to 40 years onwards in healthy aging, where some of the cognitive processes start to decline from 30 onwards (i.e., processing speed), while others mainly decline after the age of 55 years (i.e., verbal memory) [27]. This protocol paper concerns four waves which encompass three cohorts of people. Please note that in Wave 1 and 2, we did not have

For peer review only

funding yet for an accelerated longitudinal study. This means that these Waves were originally set up as independent studies. Currently, we are in the third Wave of assessments (May 2020). The time frame of data-collections is as follows: Wave 1 March 2012 until July 2014 (Cohort 1); Wave 2 December 2015 to December 2016 (Cohort 2); Wave 3 September 2018 until +/- October 2020 (Cohort 3); Wave 4 November 2020 until +/- December 2021. At the time of writing the current protocol, the COVID-19 pandemic happened. This did already affect the exact timing of the Wave 3 and might also affect the timing of Wave 4. but currently we do not know all exact consequences. One of the consequences which already occurred is that not all ADHD participants could be recruited during the time frame of Wave 3, so approximately 50% of the participants of this specific group need to be recruited during Wave 4. We, for now, consider these participants to be part of Cohort 3 as this data will be collected close to the end date of Wave 3 and, therefore, do not need to be considered a separate cohort. Please see Figure 1 and 2 for a visual depiction of the different waves and cohorts.

-Insert Figure 1 here-

Procedure and setting

After potential participants showed interest in participation, they received a study information package. Only when we received informed consent, people were actually included in the study. Please note that participants of Wave 1 were originally recruited for a cross-sectional study only, but the majority was asked to indicate whether we could contact them for future studies. Approximately 80% of the ASC group and approximately 40% of the COM group granted such permission. The Wave 1 participants between 30 and 80 years of age who gave an affirmative answer were partly recruited for Wave 2, but again for this specific Wave only. Of the newly recruited participants at Wave 2 approximately 90% of the ASC group and

For peer review only - http://bmjopen.bmj.com/site/about/guidelines.xhtml

approximately 65% of the COM group granted permission to contact them for future studies. At Wave 3 participants were asked to participate in both Wave 3 and 4, meaning that they needed to give consent to take part in two measurements at two distinct time-points with a one- to two-year interval. In each Wave participants received a series of questionnaires (on paper or via internet, see Table 1 for details). The first selection (see inclusion/exclusion criterion) is based on the first series of questionnaires (e.g., socio-demographics, diagnostic information, Autism spectrum Quotient (AQ), AD(H)D-SR)) participants filled out. If participants meet the inclusion criteria for the subtyping study, they fill out the second series of questionnaires. When based on the collected questionnaire and interview data participants also meet the first series of inclusion criteria for the cognitive study (Wave 1, 3, and 4), they are invited for a face-to-face session at the university, or closer to the participant's home upon request in order to reduce attrition rates as much as possible. In this first face-to-face session, the Autism Diagnostic Observation Scale (ADOS or ADOS-2; T1) or *Nederlands Interview ten behoeve van Diagnostiek Autismespectrumstoornis bij volwassenen* (Dutch Interview for assessment of autism spectrum disorders in adults; NIDA; T2), the International Neuropsychiatric Interview (MINI), Mini Mental State Examination (MMSE), and Wechsler Adult Intelligence Scale (WAIS) subtests are administered when it concerned autistic adults, but the ADOS(-2) (or NIDA) is not administered in the COM groups (see Table 2). In Wave 3 and 4, a questionnaire called the menopause rating scale (MRS) is added to this first session in a subset of the participants. If the autistic participants still meet inclusion criteria, a second face-to-face session will take place in which the cognitive tests will be administered (see for the specific tests Table 3). For the non-autistic/non-ADHD comparison (COM) group the two face-to-face sessions were combined on request. This implies that in theory in this group it could be that subgroup of COM participants did not meet the inclusion criteria for the cognitive section of the study, but still did do the cognitive tasks. Due to the COVID-19 pandemic the first face-to-face session was transferred to an online setting, but for the ADOS

For peer review only

and for the cognitive testing such a transition is not possible so these sessions remain face-to-face. In each Wave where cognitive testing is part of the protocol, there were 16 predetermined test orders which are counterbalanced across participants. Both sessions took place in a quiet distraction-free room in one or two sittings (each approximately 2 hours). In Wave 1 control participants received a small additional fee for participating. In Wave 3 and 4 all participants will also receive additional financial compensation for filling out the questionnaires as well as the face-to-face sessions (max. 30 euros). In all Waves travel costs are reimbursed. Some participants of Wave 1 were also invited to take part in an MRI study [28–30], but that study is not included in this protocol.

Study population and recruitment

For Cohort 1 individuals between 18 and 80 years were recruited (recruitment Wave 1), for Cohort 2 individuals over 45 years were recruited (recruitment Wave 2) , for Cohort 3 individuals between 30 and 90 years were recruited (recruitment Wave 3). Individuals with ASC and/or ADHD were recruited through several mental health institutions across the Netherlands, by means of advertisement on client organization websites and newsletters, and through social media (i.e., Twitter and LinkedIn). Comparison participants were recruited through social media and the personal networks of the researchers and participants. In order to ensure an equal proportion of participants across the whole age range and in order to match the comparison group with the ASC group, people are recruited within age bins of 10 years of age and with a sex distribution of 2:1 (males:females).

Inclusion criteria for all participants were: 1) no intellectual disability, as reported on questionnaires, or an IQ >70 as estimated using two subtests of the Wechsler Adult Intelligence Scale III or IV-NL (WAIS-III; [31] WAIS-IV-NL; [32,33]); and (2) sufficient understanding of Dutch language (i.e. at least one Dutch parent, Dutch was spoken in their family or when participants fill in more than 90% of the questionnaires).

For peer review only

An additional inclusion criterion for the ASC or ADHD group was a prior clinical ASD and/or ADHD diagnosis according the DSM- IV or DSM-5 [1,34]. For the COM group the exclusion criteria were: 1) clinical diagnosis of ASC or ADHD in themselves or direct family members; 2) an elevated score on the AQ >32 (AQ; [35] or an elevated score on ADHD-SR ≥ 6 [36]; and/or 3) reporting more than one psychosis.

For cognitive testing additional inclusion criteria were applied. All participants have to have 1) MMSE >18 [37]]; 2) no history of neurological disorders (e.g., epilepsy, stroke, cerebral contusion); 3) no current alcohol or drugs dependency; and 4) no more than 1 psychosis.

Measures

All measures and the waves at which they were administered are depicted in Tables 1, 2, and 3.

Questionnaires.

First a series of general questions were asked regarding, among others, birth year and month, gender, education, diagnostic history, ASC/ADHD in family members, and co-occurring conditions. Second, standard questionnaires are included which assessed several domains of functioning, namely: a) ASC domains including general autism traits [AQ;[35,38]], interpersonal reactivity [IRI; [39,40]], sensory sensitivities [SSQ;[15,41]]; b) mental health including ADHD characteristics [ADHD-SR; [36]]; general mental health problems (SCL-90; [42,43]; c) Physical health [Health questionnaire; [44]] and physical activity [IPAQ; [45]]; d) Well-being [Happiness; [46]] and Quality of life [WHOQoL-Bref; [47,48]]; e) Stress and worries [Worry Scale/ Fear Questionnaire; [49,50]] and Stress and self-control [Pearlin Mastery Scale; [51]]; f) subjective cognitive complaints [CFQ; [52,53]]; g) important life

For peer review only

events [Brugha;[54] [55]]; h) emotions [PANAS; [56,57]; i) social network [Close Persons Questionnaire (CPQ); [58]]; j) camouflaging of autistic traits [CAT-Q; [59,60]]; h) menopausal complaints [MRS;[61]]. The measures were chosen based on the following criteria: a) valid to be used in aging and/or autism related studies; b) easy to administer; and c) inexpensive to administer. Moreover, an additional inclusion criterion for the measures specifically included for Aim 2 is that these instruments measure factors which have predictive value for aging related outcome measures of interest for autistic adults (i.e., cognition, psychological complaints, and well-being).

-Insert Table 1 around here-

Diagnostic assessment

Global cognitive functioning was assessed with the MMSE; [37][62] and general intelligence was estimated by two subtests [Vocabulary and Matrix Reasoning] of the WAIS-III Wave 1/WAIS-IV Wave 3 and 4 [31][32,33]). Autistic traits were assessed with either the ADOS [63,64] or the NIDA [65], dependent on the measurement the participant was performing (i.e. ADOS was administered at the first measurement moment (T1) for each of the autistic participants and the NIDA at subsequent moments (T2). Psychiatric comorbidity was assessed with the MINI [66,67] in all participants independent of group.

-Insert Table 2 around here-

Cognitive assessment

Participants completed a comprehensive battery of cognitive assessments in the following domains; a) Memory, including Episodic verbal memory [Rey auditory verbal learning task

For peer review only

(RAVLT); [68]], Episodic visual memory [Wechsler memory test- III (WMS-III; [69])], Prospective memory [Amsterdam breakfast task;[70,71] in-house development author AG], Working memory [N-Back; [13]]; b) Social cognition [Faux pas; Theory of Mind;[72,73]]; c) Processing speed [2-choice reaction time (2-CRT)[15]];d) Executive functioning including generativity [Dutch version (DAT) of the Controlled Word Association Test (COWAT) [74]], planning [Tower of London; [75]], Inhibition [Simon task [14], Go/Nogo task;[in-house development by AGL], Flexibility [trail making test (TMT) A&B; [76]];d) attention (oddball; in-house development by AGL).

-Insert Table 3 around here-

Data management and quality control.

Following the institutional and national guidelines the data was pseudonymized and encrypted during and after data collection. For quality control the data of all Waves was entered by participating master students and research assistants and between 10-100% was checked by other master students, research assistants, and the PhD students involved in Wave 1, 3, and 4. The initial percentage checked depended on who entered the data for the first time and whether mistakes were discovered. When mistakes were discovered in the first checked subset, there was a 100% check.

Patient and Public Involvement

Before the start of Wave 3, we formalized the involvement of the major stakeholder group, the autistic adults. We meet a group of four older autistic adults at least three times a year for a four-year period in which we discuss, for example, study designs, questionnaires, letters, tasks and task instructions, study results, and the dissemination of these results. They are paid

For peer review only

for their contribution. In earlier waves the involvement of the stakeholders was not formally organized.

Data analysis plan and Power.

The analyses of Wave 1 are described in detail in the published papers [13–15,77,78]. Data analysis plan related to predominantly Wave 3 data are registered on As Predicted. For example, a detailed statistical analysis plan is provided for our cross-sectional replication of our previous cognition findings at https://aspredicted.org/blind.php?x=at2iq2. We also provided a detailed analysis plan for subgrouping on T1 Wave 2 and Wave 3 data by means of community detection at https://aspredicted.org/blind.php?x=hu4ey6. All final and detailed data analysis plans regarding future manuscripts based on the combined data across the different waves will be preregistered in detail on AsPredicted or similar platforms before data collection of Wave 4 ends. In these plans we will also include attrition rate handling, outlier detection, and how we will deal with item-level, instrument-level, or time-point-level missing data depending on the question at hand.

In order to determine whether we can replicate the cross-sectional findings with an accelerated longitudinal dataset (i.e., Aim 1), we will for example apply dynamic growth models, to fit the developmental (aging) shape of each cognitive process. The proposed set-up ensures that individual data points can be plotted against time (for an ASC example see [79]) and that we can actually build a linear mixed model of change in which we can model individual change, potential cohort effects, and potential measurement effects. Moreover, these techniques can provide information on whether growth curves differ shape for different cognitive subdomains. For these analyses we primarily will use data points for those participants of whom we have cognitive data at two timepoints (T1 and T2). For Aim 2 we will mainly use a bottom-up approach, were community detection will be used in order to form subgroups for which we perform an independent replication, focus on external

For peer review only

validation on T1, longitudinal stability of subgrouping and predictive validity from T1 to T2. While we predict that these T1-based subtypes will differ in, for example, their cognitive profiles, comorbidities, and subjective well-being at both T1 and T2. there is no guarantee that these subtypes will be the subtypes that actually differ in their cognitive aging process. Therefore, next to the aforementioned approach, we will use a second top-down approach in which the cognitive change will be taken as the starting point. For this second approach we did not power the study and this approach is more explorative in nature but in this way, we can focus on the heterogeneity regarding cognitive aging. First, we will determine the amount of cognitive decline one shows. This will be determined in the subset of participants for which we have cognitive data by using multivariate normative comparison (MCN) to quantify the severity of cognitive impairment. MCN can provide, next to a dichotomous measure, a continuous measure which reflects the degree of cognitive deviation. Next, we will explore which measures predict whether or not someone will show a cognitive decline, stays the same or has an increased cognitive performance.

In order to detect a (continuous) change with the planned statistical methods on the included cognitive measures (Aim 1), we will need a sample size of +/- 100 participants in the ASC group and 85 participants in the COM group at two time points when assuming an effect size of .20 and power of .80 [26,79]. In order to reach at least these numbers, we will need to recruit more T1 participants than needed for these analyses to a) be able to match on age and sex; and b) to assure sufficient participants at T2 when considering attrition rates of 30 (ASC group) to 50% (COM group). The inclusion criteria (see earlier) are the strictest for the cognitive study, which implies we need to oversample when collecting the questionnaire data as participants needed for Aim 1, will be a subset of the participants needed for Aim 2. Required sample sizes for Aim 1 and 2 differ. For Aim 2 (subtyping) it is of importance that we include a sufficiently large number of participants to have a) a training dataset to determine subgroups based on 14 variables of 8 different questionnaires and b) a test dataset

For peer review only

to determine whether the subgroup solution based on the same 14 variables is similar to the training dataset. This data needs to be collected in both Wave 2 (training dataset) and Wave 3 (test dataset) combined. Moreover, to test the temporal stability of the subgroups obtained, we require T2 data from different cohorts of participants (Wave 3 and Wave 4). We expect subgroups both within the ASC group, and in a combined sample of ASC + COM. Through simulation, we find that to reliably recover three subgroups, we require at least 160 participants. Therefore, we need to collect data from a minimum of 240 participants at T2 (160 ASC, 80 COM, see also Figure 1 and 2). In addition, we collect a minimum of 30 participants with ADHD at T2. The attrition rate from Wave 3 to Wave 4 is expected to be lower (=/- 40% across ASC and COM groups) for the questionnaire data given that people do not need to come to the lab. For the ADHD group we estimated the attrition rate to be +/- 50%. Please note that we will compare those individuals who were successfully followed up to those lost to follow up on the following variables: Autism and ADHD severity (based on AQ and ADHD-RS), age, estimated IQ, and the presence comorbid conditions (both psychiatric and somatic) in order to examine the impact of attrition on our findings.

-Insert Figure 2 here-

Ethics and dissemination.

This study is low risk in terms of ethical considerations, since participants will not receive an intervention or rules to live by. Moreover, there is no medical risk associated with participation. Apart from time and energy investment, there is no “burden” associated with participation. We chose to exclude those questions in the interview that were meant to determine whether there are current suicidal thoughts as affirmative answers as such questions could give raise to an ethical dilemma. Moreover, while we do use questionnaires and interviews which are often used in clinical practice during ASC assessment, these

For peer review only

instruments cannot be used as standalone diagnostic instruments. Hence, based on these instruments one cannot conclude for clinical purposes whether someone received an unjustified ASC diagnosis or whether they should have an additional diagnosis of a co-occurring condition. Consequently, we did not communicate about the individual outcomes with participants of these tests in accordance with the rules as set by the local ethical review board. Ethical approval for this study was obtained from the local ethical review board of the department of Psychology of the University of Amsterdam (Wave 1 2011-PN-1952 and 2013-PN-2668, Wave 2 2015-BC-4270, Wave 3 and 4 2018-BC-9285, see also supplementary material). Local research committees of the participating clinical centers will often need to give additional approval before one can recruit participants at this specific center, but will not need to conduct an additional ethical review given that the ethical approval is already covered by the ethical review board of our university. Thus, all procedures performed were in accordance with the ethical standards of the institutional ethical and research committees and with the 1964 Helsinki declaration and its later amendments or comparable ethical standards.

Study results will be published in peer-reviewed (inter)national journals (open access), and presented at conferences. Participants of all waves will receive information regarding the outcomes on a group level and receive a newsletter throughout the duration of the study when they consented to receive such information. Furthermore, these yearly newsletters are also published on our website (dutcharc.nl) and at the end of this overarching project a conference will be organized to which different stakeholders (participants, autistic adults and their relatives, clinicians, and researchers) will be invited. In both 2016 (main focus participants and clinicians of participating clinical centers) and 2018 (all aforementioned groups) such conferences were already successfully organized.

For peer review only

Contributorship statement

All authors declare that they contributed to the content of this manuscript and commented on subsequent versions of the manuscript and were involved in parts of the design of the overall study. Each of the authors comments on each other's statistical analyses plans before it is submitted to AsPredicted/Open Science Framework. Please see Table 4 for an easy overview of the contributors of each of the authors and of people who were involved in an earlier stage (see also acknowledgements). In short, Hilde M. Geurts |(H.M.G) secured funding, was responsible for the overall design related to each of the waves and regarding the initial set up for both the subtyping as well as cognition study across waves, is involved in the set up for the statistical analyses, and wrote, together with Annabeth P. Groenman (APG), the first draft of this manuscript. Joost Agelink van Rentergem (JAR) and Tulsi Radhoe (TAR) were involved in the study design and statistical analysis plan of the subgrouping study. Carolien Torenvliet (CT) and APG were involved in the study design and statistical analysis plan of the cognition study. TAR, CT, and Wikke J. Van der Putten (WvdP) are collecting the data in Wave 3 and 4 and, together with HMG and an external collaborator, involved in the statistical analysis plans for all questions related to camouflaging. TAR, CT, and WvdP are double checking all data, and APG and HMG are supervising CT, JAR and HMG are supervising TAR, and HMG is supervising WvdP together with an external collaborator who is not involved in the setup of the current study or in the data collection.

-Please insert Table 4 here-

Funding statement

This study was funded by the Grant Sponsor is the Netherlands Organization for Scientific Research (NWO); VIDI Grant (grant number: 452–10-003 and VICI Grant (grant number: 453–16-006) awarded to HMG. This funding organization had no involvement in study design, analysis, or interpretation of the data but does require open access publication of the findings. Both project grants were reviewed by three (VIDI) to five (VICI) anonymous and independent researchers.

For peer review only

Data sharing statement

Analyzed data is directly available when requested from the first author during peer review and after publication by non-commercial researchers affiliated with a university. Within five years after the planned future publications, data will become available upon request for non-commercial researchers affiliated with a university to answer additional questions via a repository of the University of Amsterdam.

Competing interest statement

None of the authors have competing interest relevant to the work to report.

Acknowledgements

We want to thank the past, current, and future participants. The students who helped with the data collections as previous, current, and future research assistants. We also thank the institutions and organizations who helped/are helping with recruitment. We are especially grateful by the time and advice provided by the older autistic adults in our think tank. We mention two people by name Anne Geeke Lever (AGL) and Iuno Groot (IG) as AGL was as first PhD student on a related project instrumental for the set up and data collection of Wave 1 and IG, as research assistant, for the data collection of Wave 2. Therefore, we thank both AGL and IG for their involvement regarding the first studies related to autism and aging on which we build the future measurements waves.

For peer review only

References

1 American Psychiatric Association. *Diagnostic and Statistical Manual of Mental Disorders*. Fifth Edition. American Psychiatric Association 2013. doi:10.1176/appi.books.9780890425596

2 Brugha TS, McManus S, Smith J, *et al.* Validating two survey methods for identifying cases of autism spectrum disorder among adults in the community. *Psychol Med* 2011;:1–10. doi:S0033291711001292 [pii] 10.1017/S0033291711001292

3 Chiarotti F, Venerosi A. Epidemiology of Autism Spectrum Disorders: A Review of Worldwide Prevalence Estimates Since 2014. *Brain Sci* 2020;**10**:274. doi:10.3390/brainsci10050274

4 Happé F, Frith U. Annual Research Review: Looking back to look forward – changes in the concept of autism and implications for future research. *J Child Psychol Psychiatry* 2020;**61**:218–32. doi:10.1111/jcpp.13176

5 Howlin P, Savage S, Moss P, *et al.* Cognitive and language skills in adults with autism: a 40-year follow-up. *J Child Psychol Psychiatry* 2014;**55**:49–58. doi:10.1111/jcpp.12115

6 Lombardo MV, Lai M-C, Baron-Cohen S. Big data approaches to decomposing heterogeneity across the autism spectrum. *Mol Psychiatry* 2019;**24**:1435–50. doi:10.1038/s41380-018-0321-0

7 Veenstra-VanderWeele J, Blakely RD. Networking in Autism: Leveraging Genetic, Biomarker and Model System Findings in the Search for New Treatments. *Neuropsychopharmacology* 2012;**37**:196–212. doi:10.1038/npp.2011.185

8 Mukaetova-Ladinska EB, Perry E, Baron M, *et al.* Ageing in people with autistic spectrum disorder: Ageing in people with autistic spectrum disorder. *Int J Geriatr Psychiatry* 2012;**27**:109–18. doi:10.1002/gps.2711

9 Roestorf A, Bowler DM, Deserno MK, *et al.* "Older Adults with ASD: The Consequences of Aging." Insights from a series of special interest group meetings held at the International Society for Autism Research 2016–2017. *Res Autism Spectr Disord* 2019;**63**:3–12. doi:10.1016/j.rasd.2018.08.007

10 Happé F, Charlton RA. Aging in autism spectrum disorders: a mini-review. *Gerontology* 2012;**58**:70–8. doi:10.1159/000329720

11 Lai M-C, Baron-Cohen S. Identifying the lost generation of adults with autism spectrum conditions. *Lancet Psychiatry* 2015;**2**:1013–27. doi:10.1016/S2215-0366(15)00277-1

12 Geurts HM, Vissers ME. Elderly with autism: executive functions and memory. *J Autism Dev Disord* 2012;**42**:665–75. doi:10.1007/s10803-011-1291-0

13 Lever AG, Werkle-Bergner M, Brandmaier AM, *et al.* Atypical working memory decline across the adult lifespan in autism spectrum disorder? *J Abnorm Psychol* 2015;**124**:1014–26. doi:10.1037/abn0000108

For peer review only - http://bmjopen.bmj.com/site/about/guidelines.xhtml

14 Lever AG, Ridderinkhof KR, Marsman M, *et al.* Reactive and proactive interference control in adults with autism spectrum disorder across the lifespan. *Dev Psychol* 2017;**53**:379–95. doi:10.1037/dev0000219

15 Lever AG, Geurts HM. Age-related differences in cognition across the adult lifespan in autism spectrum disorder. *Autism Res Off J Int Soc Autism Res* 2016;**9**:666–76. doi:10.1002/aur.1545

16 Demetriou EA, Lampit A, Quintana DS, *et al.* Autism spectrum disorders: a meta-analysis of executive function. *Mol Psychiatry* 2018;**23**:1198–204. doi:10.1038/mp.2017.75

17 Davids RCD, Groen Y, Berg IJ, *et al.* Executive Functions in Older Adults With Autism Spectrum Disorder: Objective Performance and Subjective Complaints. *J Autism Dev Disord* 2016;**46**:2859–73. doi:10.1007/s10803-016-2831-4

18 Geurts HM, Pol SE, Lobbestael J, *et al.* Executive Functioning in 60+ Autistic Males: The Discrepancy Between Experienced Challenges and Cognitive Performance. *J Autism Dev Disord* Published Online First: 17 January 2020. doi:10.1007/s10803-020-04368-9

19 Braden BB, Smith CJ, Thompson A, *et al.* Executive function and functional and structural brain differences in middle-age adults with autism spectrum disorder. *Autism Res Off J Int Soc Autism Res* 2017;**10**:1945–59. doi:10.1002/aur.1842

20 Powell PS, Klinger LG, Klinger MR. Patterns of Age-Related Cognitive Differences in Adults with Autism Spectrum Disorder. *J Autism Dev Disord* 2017;**47**:3204–19. doi:10.1007/s10803-017-3238-6

21 Tse VWS, Crabtree J, Islam S, *et al.* Comparing Intellectual and Memory Abilities of Older Autistic Adults with Typically Developing Older Adults Using WAIS-IV and WMS-IV. *J Autism Dev Disord* 2019;**49**:4123–33. doi:10.1007/s10803-019-04122-w

22 Walsh MJM, Baxter LC, Smith CJ, *et al.* Age group differences in executive network functional connectivity and relationships with social behavior in men with autism spectrum disorder. *Res Autism Spectr Disord* 2019;**63**:63–77. doi:10.1016/j.rasd.2019.02.008

23 McPartland JC. Considerations in biomarker development for neurodevelopmental disorders: *Curr Opin Neurol* 2016;**29**:118–22. doi:10.1097/WCO.0000000000000300

24 Cholemkery H, Medda J, Lempp T, *et al.* Classifying Autism Spectrum Disorders by ADI-R: Subtypes or Severity Gradient? *J Autism Dev Disord* 2016;**46**:2327–39. doi:10.1007/s10803-016-2760-2

25 Rommelse NNJ, Geurts HM, Franke B, *et al.* A review on cognitive and brain endophenotypes that may be common in autism spectrum disorder and attention-deficit/hyperactivity disorder and facilitate the search for pleiotropic genes. *Neurosci Amp Biobehav Rev* 2011;**35**:1363–96.

For peer review only

26 Fabbri E, An Y, Zoli M, *et al.* Association Between Accelerated Multimorbidity and Age-Related Cognitive Decline in Older Baltimore Longitudinal Study of Aging Participants without Dementia. *J Am Geriatr Soc* 2016;**64**:965–72. doi:10.1111/jgs.14092

27 Hedden T, Gabrieli JDE. Insights into the ageing mind: a view from cognitive neuroscience. *Nat Rev Neurosci* 2004;**5**:87–96.

28 Bathelt J, Koolschijn PC, Geurts HM. Age-variant and age-invariant features of functional brain organization in middle-aged and older autistic adults. *Mol Autism* 2020;**11**:9. doi:10.1186/s13229-020-0316-y

29 Koolschijn PCMP, Caan MWA, Teeuw J, *et al.* Age-related differences in autism: The case of white matter microstructure. *Hum Brain Mapp* 2017;**38**:82–96. doi:10.1002/hbm.23345

30 Koolschijn PCMP, Geurts HM. Gray Matter Characteristics in Mid and Old Aged Adults with ASD. *J Autism Dev Disord* 2016;**46**:2666–78. doi:10.1007/s10803-016-2810-9

31 Wechsler. WAIS-III. *San Antonio Psychol Corp* 1997.

32 Wechsler. WAIS-IV. 2008.

33 Uterwijk J. WAIS-III Nederlandstalige bewerking. Technische handleiding. 2000.

34 American Psychiatric Association. *Diagnostic and Statistical Manual of Mental Health Disorders*. Washington DC: American Psychiatric Association 1994.

35 Baron-Cohen, Wheelwright S, Skinner R, *et al.* The autism-spectrum quotient (AQ): evidence from Asperger syndrome/high-functioning autism, males and females, scientists and mathematicians. *J Autism Dev Disord* 2001;**31**:5–17. doi:10.1023/a:1005653411471

36 Kooij JS, Buitelaar JK, FURER JW, *et al.* Internal and external validity of attention-deficit hyperactivity disorder in a population-based sample of adults. *Psychol Med* 2005;**35**:817–827.

37 Folstein MF, Folstein SE, McHugh PR. "Mini-mental state": a practical method for grading the cognitive state of patients for the clinician. *J Psychiatr Res* 1975;**12**:189–98.

38 Hoekstra RA, Bartels M, Cath DC, *et al.* Factor structure, reliability and criterion validity of the Autism-Spectrum Quotient (AQ): a study in Dutch population and patient groups. *J Autism Dev Disord* 2008;**38**:1555–66. doi:10.1007/s10803-008-0538-x

39 Davis MH. A multidimensional approach to individual differences in empathy. 1980.

40 De Corte K, Buysse A, Verhofstadt LL, *et al.* Measuring empathic tendencies: Reliability and validity of the Dutch version of the Interpersonal Reactivity Index. *Psychol Belg* 2007;**47**:235–260.

For peer review only

41 Minshew NJ, Hobson JA. Sensory sensitivities and performance on sensory perceptual tasks in high-functioning individuals with autism. *J Autism Dev Disord* 2008;**38**:1485–98.

42 Arrindell W, Ettema J. Symptom checklist: Handleiding bij een multidimensionale psychopathologie-indicator SCL-90. 2005.

43 Derogatis LR. Symptoms checklist-90. *Adm Scoring Proced Man Revis Version* 1977.

44 Central Bureau of Statistics (CBS). Health Interview Questionnaire. *Heerlen Neth* 1989;**Central Bureau of Statistics**.

45 Craig CL, Marshall AL, Sjöström M, *et al.* International physical activity questionnaire: 12-country reliability and validity. *Med Sci Sports Exerc* 2003;**35**:1381–1395.

46 Bartels M, Boomsma DI. Born to be Happy? The Etiology of Subjective Well-Being. *Behav Genet* 2009;**39**:605–15. doi:10.1007/s10519-009-9294-8

47 Whoqol Group. Development of the World Health Organization WHOQOL-BREF quality of life assessment. *Psychol Med* 1998;**28**:551–558.

48 Trompenaars FJ, Masthoff ED, Van Heck GL, *et al.* Content validity, construct validity, and reliability of the WHOQOL-Bref in a population of Dutch adult psychiatric outpatients. *Qual Life Res* 2005;**14**:151–160.

49 Marks IM, Mathews AM. Brief standard self-rating for phobic patients. *Behav Res Ther* 1979;**17**:263–267.

50 Wisocki PA, Handen B, Morse CK. The Worry Scale as a measure of anxiety among homebound and community active elderly. *Behav Ther* 1986.

51 Pearlin LI, Schooler C. The structure of coping. *J Health Soc Behav* 1978;:2–21.

52 Broadbent DE, Cooper PF, FitzGerald P, *et al.* The cognitive failures questionnaire (CFQ) and its correlates. *Br J Clin Psychol* 1982;**21**:1–16.

53 Merckelbach H, Muris P, Nijman H, *et al.* Self-reported cognitive failures and neurotic symptomatology. *Personal Individ Differ* 1996;**20**:715–724.

54 Hull L, Mandy W, Lai M-C, *et al.* Development and validation of the camouflaging autistic traits questionnaire (CAT-Q). *J Autism Dev Disord* 2019;**49**:819–33.

55 Brugha TS, McManus S, Bankart J, *et al.* Epidemiology of autism spectrum disorders in adults in the community in England. *Arch Gen Psychiatry* 2011;**68**:459–465.

56 Peeters F, Ponds R, Vermeeren MTG. Affectiviteit en zeltbeoordeling van depressie en angst. *Tijdschr Voor Psychiatr* 1996;**38**:240–250.

57 Watson D, Clark LA, Tellegen A. Development and validation of brief measures of positive and negative affect: the PANAS scales. *J Pers Soc Psychol* 1988;**54**:1063.

For peer review only

58 Stansfeld S, Marmot M. Deriving a survey measure of social support: the reliability and validity of the Close Persons Questionnaire. *Soc Sci Med* 1992;**35**:1027–1035.

59 Agelink van Rentergem J, Radhoe T, Geurts HM. A Dutch translation of the CAT-Q.

60 Hull L, Mandy W, Lai M-C, *et al.* Development and validation of the camouflaging autistic traits questionnaire (CAT-Q). *J Autism Dev Disord* 2019;**49**:819–833.

61 Greene JG. Measuring the symptom dimension of quality of life: General and menopause-specific scales and their subscale structure. *Horm Replace Ther Qual Life* 2002.

62 Kok R, Verhey F. Dutch translation of the mini mental state examination (Folstein et al., 1975). *Altrecht GGZ* 2002.

63 Bildt A, Greaves-Lord K, De Jonge M. ADOS-2 Nederlandse bewerking.[ADOS-2 Dutch Adaptation]. *Amst Neth Hogreve Uitgevers* 2013.

64 Lord C, Risi S, Lambrecht L, *et al.* The Autism Diagnostic Observation Schedule—Generic: A standard measure of social and communication deficits associated with the spectrum of autism. *J Autism Dev Disord* 2000;**30**:205–223.

65 Vuijk R. *Nederlands Interview ten behoeve van Diagnostiek Autismespectrumstoornis bij Volwassenen (NIDA)*. Amsterdam: Boom Uitgevers 2014.

66 Sheehan DV, Lecrubier Y, Sheehan KH, *et al.* The validity of the Mini International Neuropsychiatric Interview (MINI) according to the SCID-P and its reliability. *Eur Psychiatry* 1997;**12**:232–241.

67 Van Vliet I, Leroy H, Van Megen H. De MINI internationaal neuropsychiatrisch interview, nederlandse versie 5.0. 0. *Leiden Leiden Univ Med Cent Dep Psychiatry* 2000.

68 Rey A. L'examen psychologique dans les cas d'encéphalopathie traumatique.(Les problems.). *Arch Psychol* 1941.

69 Wechsler D. Wechsler memory scale (WMS-III)(Vol. 14). *San Antonio TX Psychol Corp* 1997.

70 Altgassen M, Koban N, Kliegel M. Do Adults with Autism Spectrum Disorders Compensate in Naturalistic Prospective Memory Tasks? *J Autism Dev Disord* 2012;**42**:2141–51. doi:10.1007/s10803-012-1466-3

71 Craik FI, Bialystok E. Planning and task management in older adults: Cooking breakfast. *Mem Cognit* 2006;**34**:1236–49.

72 Spek AA, Scholte EM, Van Berckelaer-Onnes IA. Theory of Mind in Adults with HFA and Asperger Syndrome. *J Autism Dev Disord* 2010;**40**:280–9. doi:10.1007/s10803-009-0860-y

For peer review only

73 Stone VE, Baron-Cohen S, Knight RT. Frontal Lobe Contributions to Theory of Mind. *J Cogn Neurosci* 1998;**10**:640–56. doi:10.1162/089892998562942

74 Luteijn F, Barelds DPH. *GIT2: Groninger Intelligentie Test 2*. Harcourt Test Publishers 2004.

75 Shallice T. Specific impairments of planning. *Philos Trans R Soc Lond B Biol Sci* 1982;**298**:199–209.

76 Reitan RM, Wolfson D. TheHalslead-Reitan Neuropsychological Jesi Battery: theory and interpretai ion. 1985.

77 Lever AG, Geurts HM. Psychiatric Co-occurring Symptoms and Disorders in Young, Middle-Aged, and Older Adults with Autism Spectrum Disorder. *J Autism Dev Disord* 2016;**46**:1916–30. doi:10.1007/s10803-016-2722-8

78 Lever AG, Geurts HM. Is Older Age Associated with Higher Self- and Other-Rated ASD Characteristics? *J Autism Dev Disord* 2018;**48**:2038–51. doi:10.1007/s10803-017-3444-2

79 Moerbeek M. The Effects of the Number of Cohorts, Degree of Overlap Among Cohorts, and Frequency of Observation on Power in Accelerated Longitudinal Designs. *Methodology* 2011;**7**:11–24. doi:10.1027/1614-2241/a000019

For peer review only

Tables and Figures

Table 1. Timing and use of questionnaires

<table>
<tr><td colspan="2">Measures</td><td colspan="4">Waves</td><td colspan="2">Study focus</td></tr>
<tr><td>Instrument</td><td>Construct</td><td>1</td><td>2</td><td>3</td><td>4</td><td>Aim 1</td><td>Aim 2*</td></tr>
<tr><td>General</td><td>Descriptives</td><td style="background:#c0c0c0"></td><td style="background:#c0c0c0"></td><td style="background:#c0c0c0"></td><td style="background:#c0c0c0"></td><td>INCL</td><td>INCL/SUB</td></tr>
<tr><td>AQ- self</td><td>Autism traits</td><td style="background:#c0c0c0"></td><td style="background:#c0c0c0"></td><td style="background:#c0c0c0"></td><td style="background:#c0c0c0"></td><td>INCL</td><td>SUB</td></tr>
<tr><td>AQ- proxy</td><td>Autism traits</td><td style="background:#c0c0c0"></td><td></td><td></td><td></td><td></td><td></td></tr>
<tr><td>IRI-self</td><td>Personal relations</td><td style="background:#c0c0c0"></td><td></td><td></td><td></td><td></td><td></td></tr>
<tr><td>IRI-proxy</td><td>Personal relations</td><td style="background:#c0c0c0"></td><td></td><td></td><td></td><td></td><td></td></tr>
<tr><td>SSQ-self</td><td>Sensory sensitivities</td><td style="background:#c0c0c0"></td><td style="background:#c0c0c0"></td><td style="background:#c0c0c0"></td><td style="background:#c0c0c0"></td><td></td><td>SUB</td></tr>
<tr><td>SSQ-proxy</td><td>Sensory sensitivities</td><td style="background:#c0c0c0"></td><td></td><td></td><td></td><td></td><td></td></tr>
<tr><td>ADHD-SR</td><td>ADHD traits</td><td style="background:#c0c0c0"></td><td style="background:#c0c0c0"></td><td style="background:#c0c0c0"></td><td style="background:#c0c0c0"></td><td>INCL</td><td></td></tr>
<tr><td>SCL-90</td><td>Mental health</td><td style="background:#c0c0c0"></td><td style="background:#c0c0c0"></td><td style="background:#c0c0c0"></td><td style="background:#c0c0c0"></td><td></td><td>VAL</td></tr>
<tr><td>Health Ques</td><td>Physical health</td><td></td><td style="background:#c0c0c0"></td><td style="background:#c0c0c0"></td><td style="background:#c0c0c0"></td><td>INCL</td><td></td></tr>
<tr><td>MRS</td><td>Menopause</td><td></td><td></td><td style="background:#c0c0c0"></td><td style="background:#c0c0c0"></td><td></td><td></td></tr>
<tr><td>CFQ</td><td>Global cognition</td><td style="background:#c0c0c0"></td><td style="background:#c0c0c0"></td><td style="background:#c0c0c0"></td><td style="background:#c0c0c0"></td><td>REP</td><td>VAL</td></tr>
<tr><td>PANAS</td><td>Global emotion</td><td></td><td style="background:#c0c0c0"></td><td style="background:#c0c0c0"></td><td style="background:#c0c0c0"></td><td></td><td>SUB</td></tr>
<tr><td>WHOQoL</td><td>Quality of Life</td><td style="background:#c0c0c0"></td><td style="background:#c0c0c0"></td><td style="background:#c0c0c0"></td><td style="background:#c0c0c0"></td><td></td><td>VAL</td></tr>
<tr><td>Happiness</td><td>Well-being</td><td></td><td style="background:#c0c0c0"></td><td style="background:#c0c0c0"></td><td style="background:#c0c0c0"></td><td></td><td></td></tr>
<tr><td>Mastery</td><td>Stress/Self-control</td><td></td><td style="background:#c0c0c0"></td><td style="background:#c0c0c0"></td><td style="background:#c0c0c0"></td><td></td><td>SUB</td></tr>
<tr><td>Worry</td><td>Stress/Worries</td><td></td><td style="background:#c0c0c0"></td><td style="background:#c0c0c0"></td><td style="background:#c0c0c0"></td><td></td><td>SUB</td></tr>
<tr><td>IPAQ</td><td>Physical activity</td><td></td><td style="background:#c0c0c0"></td><td style="background:#c0c0c0"></td><td style="background:#c0c0c0"></td><td></td><td>SUB</td></tr>
<tr><td>Brugha</td><td>Life events</td><td></td><td style="background:#c0c0c0"></td><td style="background:#c0c0c0"></td><td style="background:#c0c0c0"></td><td></td><td>SUB</td></tr>
<tr><td>CPI</td><td>Social network</td><td></td><td style="background:#c0c0c0"></td><td style="background:#c0c0c0"></td><td style="background:#c0c0c0"></td><td></td><td>SUB</td></tr>
<tr><td>CAT-Q</td><td>Camouflaging</td><td></td><td></td><td style="background:#c0c0c0"></td><td style="background:#c0c0c0"></td><td></td><td></td></tr>
</table>

For peer review only

Note. Aim 1: Arbitrate between cognitive aging hypotheses; Aim 2: Subgrouping; Gray shading indicates that a measure was administered during that Wave. ADHD-SR=Attention Deficit Hyperactivity Disorder Self Report; AQ=Autism spectrum Quotient; CFQ=Cognitive Failure Questionnaire; Brugha=Brugha questionnaire of important life events during childhood and in the past years; CPQ=Close Persons Questionnaire to measure present social support from the four most intimate persons; INCL= used for inclusion; IPAQ=International Physical Activities Questionnaire; Happiness: Perceived well-being question of the NAR; IRI= interpersonal reactivity inventory; MRS= menopause rating scale; PANAS=Positive and Negative Affect Schedule; REP= Replication; SCL-90=Symptom Checklist; SSQ=Sensory Sensitivity Questionnaire; SUB= used for subtyping; VAL= Validation; WHOQoL=World Health Organization Quality of Life.

*Please note that more than one variable can be derived from the instruments. For example, we will use 14 cluster variables for our subtyping analysis which are based on 8 different instruments.

Table 2. Timing and use of diagnostic measures

Diagnostic assessment								
	Measures		Waves				Study focus	
Type	Instrument	Construct	1	2	3	4	Aim 1	Aim 2
CNP	MMSE	Global cognition					INCL	
CNP	WAIS subtests	Estimation of intelligence					INCL	INCL
OBS	ADOS	ASC					INCL	
INT	NIDA	ASC						
INT	MINI	Mental health					INCL	VAL

Note. Gray shading indicates that a measure was administered during that Wave. ADOS= Autism Diagnostic Observation Scale; CNP= clinical neuropsychologic task; INCL= used for inclusion; INT= Interview; MINI= MINI the International Neuropsychiatric Interview; MMSE=Mini Mental State Examination; NIDA= Nederlands Interview ten behoeve van Diagnostiek Autismespectrumstoornis bij volwassenen [Dutch Interview for ASD assessment in adults]; OBS = Observation; VAL: Measure to test validity of subtypes.

For peer review only - http://bmjopen.bmj.com/site/about/guidelines.xhtml

Table 3. Timing and use of cognitive measures

Cognitive assessment						
	Measures		Waves			
Type	Instrument	Construct	1	2	3	4
CNP	RAVLT	Episodic verbal memory	■		■	■
CNP	WMS	Episodic visual memory	■		■	■
CNP	DAT & COWAT	EF (generativity)	■		■	■
CNP	TMT A & B	EF (flexibility)			■	■
CNP	ToL	EF (planning)			■	■
EXP	Go-NoGo	EF (inhibition)	■		■	■
EXP	Simon	EF (inhibition)	■			
EXP	N-back	EF (working memory)	■		■	■
EXP	2-Choice RT	Processing speed	■		■	■
CNP	Faux Pas	Social cognition (ToM)	■		■	■
EXP	Odd ball	Attention	■			
EXP	ABT	Prospective memory			■	■

Note. Gray shading indicates that a measure was administered during that Wave. ABT= Amsterdam Breakfast Task; CNP= clinical neuropsychologic task; DAT=Dutch version of the Controlled Word Association Test; EF=Executive functioning; EXP= experimental task; RAVLT=Rey Auditory Verbal Learning Test; 2-Choice RT=a simple computerized reaction time task; Simon=a computerized inhibition task; ToL= Tower of London; ToM=Theory of Mind; WMS-III=Wechsler Memory Scale 3rd edition, subtest visual reproduction.

For peer review only

Table 4 Contributions to the protocol

	HMG	AGL[a]	IG[a]	TR	JAR	CT	WvdP	APG
Funding	■							
Study design Wave 1	■	■						
Study design Wave 2	■							
Study design subgrouping (across Waves)	■			■	■			
Study design cognition (across Waves)	■					■		■
Data collection		■	■	■	■	■	■	■
Set up/ feedback statistical analyses	■			■		■	■	■
First draft of the manuscript	■							■
Feedback on subsequent versions of manuscript	■			■	■	■	■	■
Final approval of the manuscript	■			■	■	■	■	■

HMG = Hilde M. Geurts; AGL=Anne Geeke Lever; IG=Iuno Groot; TR=Tulsi Radhoe; JAR=Joost Agelink van Rentergem; CT=Carolien Torenvliet; WvdP=Wikke van der Putten; APG=Annabeth P. Groenman.

[a] were not involved in preparation of the current manuscript and design of the longitudinal study, but were involved in earlier stages of the research, see also the acknowledgements.

For peer review only

Figure captions

Figure 1. Flow charts for each separate cohort.

Note. Cohort 1 (left panel) started in March 2012, Cohort 2 (middle panel) started in December 2015. Cohort 3 started in September 2018. Data collection on Wave 4 is estimated to start end 2020, numbers reported for Wave 4 are based on expected attrition rates (i.e., 40% for questionnaires, for cognition 30% for ASC, 50% for comparisons). Participant numbers in Wave 3 are based on current inclusion in combination with our power analyses for Wave 4. From Wave 1 to Wave 2 the inclusion age increased from 20 to 30 years old (see main text). = indicates exact numbers; ≥ indicates planned numbers; ≈ estimated numbers based on expected attrition rates. Please note that we did not include information regarding the inclusion of the participants with an Attention-Deficit/ Hyperactivity Disorder diagnosis as participants of one cohort will be, due to COVID-19 be recruited across Wave 3 and 4. ASC: Autism spectrum condition; C: Cognitive tests; COM: comparison; Q: Questionnaires

Figure 2. Total number of participants seen (Wave 1 and 2) or need to be seen minimally (Wave 3 and 4) at each wave when taking into account the estimated attrition rate (see attrition) and age inclusion criterion (>30 years) at time of inclusion (see text).

Note. = indicates exact numbers; ≥ planned numbers; ≈ estimated numbers based on expected attrition rates. Please note that in this Figure no information regarding the recruitment of participants with an Attention-Deficit/ Hyperactivity Disorder diagnosis. ASC: Autism spectrum condition; C: Cognitive tests; COM: comparison; Q: Questionnaires. N.B. reported numbers at Wave 4 are based on power analyses. The number of newly recruited participants can be seen in Figure 1.

For peer review only

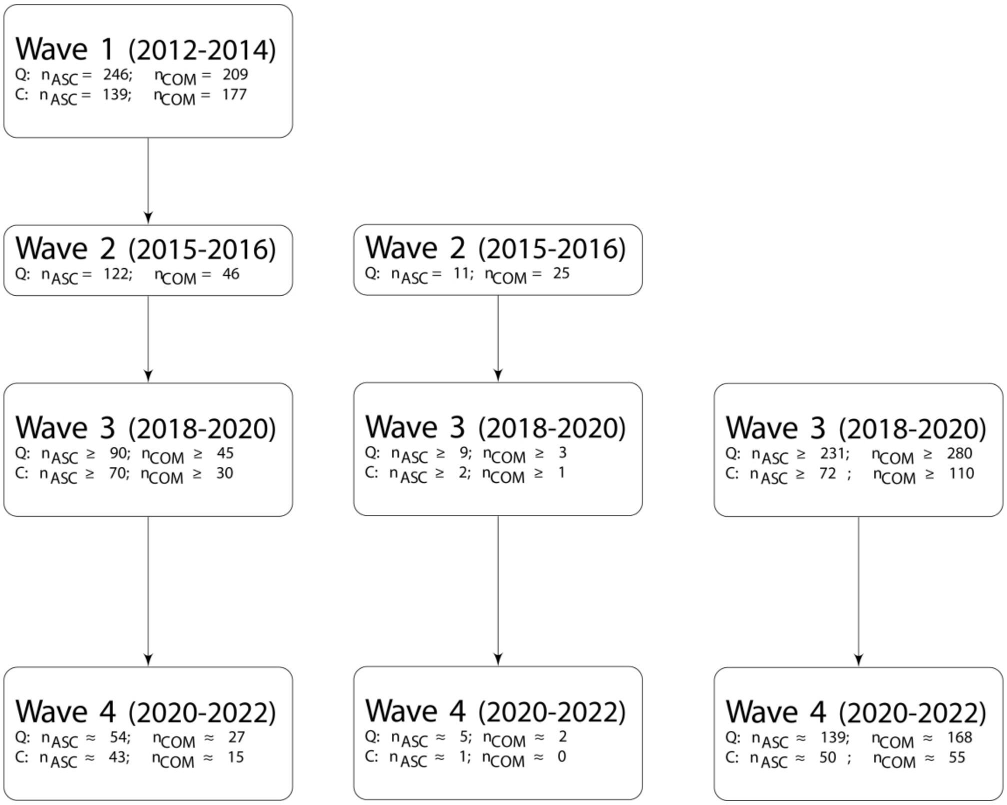

Figure 1. Flow charts for each separate cohort.

Note. Cohort 1 (left panel) started in March 2012, Cohort 2 (middle panel) started in December 2015. Cohort 3 started in September 2018. Data collection on Wave 4 is estimated to start end 2020, numbers reported for Wave 4 are based on expected attrition rates (i.e., 40% for questionnaires, for cognition 30% for ASC, 50% for comparisons). Participant numbers in Wave 3 are based on current inclusion in combination with our power analyses for Wave 4. From Wave 1 to Wave 2 the inclusion age increased from 20 to 30 years old (see main text). = indicates exact numbers; ≥ indicates planned numbers; ≈ estimated numbers based on expected attrition rates. Please note that we did not include information regarding the inclusion of the participants with an Attention-Deficit/ Hyperactivity Disorder diagnosis as participants of one cohort will be, due to COVID-19 be recruited across Wave 3 and 4. ASC: Autism spectrum condition; C: Cognitive tests; COM: comparison; Q: Questionnaires

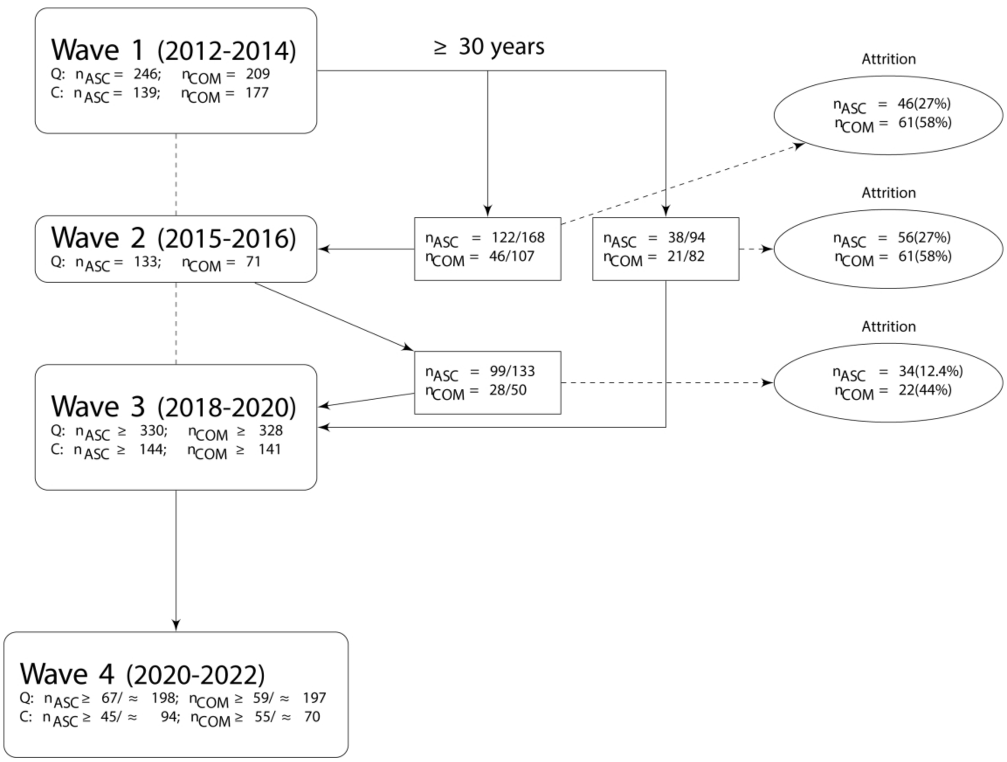

Figure 2. Total number of participants seen (Wave 1 and 2) or need to be seen minimally (Wave 3 and 4) at each wave when taking into account the estimated attrition rate (see attrition) and age inclusion criterion (>30 years) at time of inclusion (see text).
Note. = indicates exact numbers; ≥ planned numbers; ≈ estimated numbers based on expected attrition rates. Please note that in this Figure no information regarding the recruitment of participants with an Attention-Deficit/ Hyperactivity Disorder diagnosis. ASC: Autism spectrum condition; C: Cognitive tests; COM: comparison; Q: Questionnaires. N.B. reported numbers at Wave 4 are based on power analyses. The number of newly recruited participants can be seen in Figure 1.

Supplementary material

"Aging and heterogeneity regarding autism spectrum conditions: A protocol paper of an accelerated longitudinal study" Geurts, H.M., Agelink van Rentergem, J., Radhoe, T., Torenvliet, C., Putten Van der, W.J., Groenman, A.P.

Ethical approval:

Ethical approval for this study was obtained from the local ethical review board of the Psychology Department of the University of Amsterdam (Wave 1 2011-PN-1952 and 2013-PN-2668, Wave 2 2015-BC-4270, Wave 3 and 4 2018-BC-9285).

For peer review only
